# The dimerization mechanism of the N-terminal domain of spider silk proteins is conserved despite extensive sequence divergence

**DOI:** 10.1016/j.jbc.2022.101913

**Published:** 2022-04-07

**Authors:** Médoune Sarr, Kristine Kitoka, Kellie-Ann Walsh-White, Margit Kaldmäe, Rimants Metlāns, Kaspar Tārs, Alessandro Mantese, Dipen Shah, Michael Landreh, Anna Rising, Jan Johansson, Kristaps Jaudzems, Nina Kronqvist

**Affiliations:** 1 Division for Neurogeriatrics, Department of Neurobiology, Care Sciences and Society, Karolinska Institutet, Huddinge, Sweden; 2 Department of Physical Organic Chemistry, Latvian Institute of Organic Synthesis, Riga, Latvia; 3 Department of Microbiology, Tumor and Cell Biology, Karolinska Institutet, Solna, Sweden; 4 Latvian Biomedical Research and Study Centre, Riga, Latvia; 5 ZoBio BV, Leiden, The Netherlands; 6 Department of Anatomy, Physiology and Biochemistry, Swedish University of Agricultural Sciences, Uppsala, Sweden; 7 Department of Biosciences and Nutrition, Neo, Karolinska Institutet, Huddinge, Sweden

**Keywords:** protein domain, dimerization, silk assembly, spidroin, NMR structure, X-ray structure, CD, circular dichroism, ESI-MS, electrospray ionization mass spectrometry, FlSp, flagelliform spidroin, HSQC, heteronuclear single quantum coherence, MALS, multiangle light scattering, MaSp, major ampullate spidroin, MiSp, minor ampullate spidroin, MW, molecular weight, NMR, nuclear magnetic resonance, NT, N-terminal, PFG, pulsed-field gradient, SEC, size-exclusion chromatography

## Abstract

The N-terminal (NT) domain of spider silk proteins (spidroins) is crucial for their storage at high concentrations and also regulates silk assembly. NTs from the major ampullate spidroin (MaSp) and the minor ampullate spidroin are monomeric at neutral pH and confer solubility to spidroins, whereas at lower pH, they dimerize to interconnect spidroins in a fiber. This dimerization is known to result from modulation of electrostatic interactions by protonation of well-conserved glutamates, although it is undetermined if this mechanism applies to other spidroin types as well. Here, we determine the solution and crystal structures of the flagelliform spidroin NT, which shares only 35% identity with MaSp NT, and investigate the mechanisms of its dimerization. We show that flagelliform spidroin NT is structurally similar to MaSp NT and that the electrostatic intermolecular interaction between Asp 40 and Lys 65 residues is conserved. However, the protonation events involve a different set of residues than in MaSp, indicating that an overall mechanism of pH-dependent dimerization is conserved but can be mediated by different pathways in different silk types.

Spider silk has outstanding mechanical properties that surpass man-made materials and represents an attractive material for many potential applications. Orb-weaving spiders are able to synthesize up to seven types of silk for various purposes, including major ampullate, minor ampullate, flagelliform, aciniform, aggregate, tubiliform and pyriform silk (1). Each type of silk is produced in a dedicated gland from highly specialized polymer building blocks consisting of large proteins (spidroins). Dragline or major ampullate silk is the strongest silk type and makes up the framework of the web or is used as a safety line by the spider (2). Detailed studies of the morphology of the major ampullate gland, where this silk is produced, have identified three distinct regions: a narrow long tail where spidroins are synthesized, a bulb-like ampulla (sac) where spidroins are stored, and a narrowing s-shaped duct where different biochemical stimuli, including shear forces and a pH drop, convert spidroins into silk fibers during spinning (3, 4, 5, 6). The minor ampullate silk is used by the spider to strengthen the web, and the corresponding gland closely resembles the major ampullate gland although the size is smaller. The flagelliform silk is the most extensible form of silk and forms the thread in the capture spiral of the web. The flagelliform gland differs from the ampullate glands by being much smaller in size and by having a very short spinning duct (7). It is possible that the short duct of this gland provides environmental conditions different from the ampullate glands, for example a sharper or shorter pH gradient, but the biochemical events driving silk formation in the flagelliform gland have not been investigated in detail.

Spidroins harbor up to several thousand residues (8). They are made up of highly repetitive and silk-specific parts, which determine the mechanical properties of the fibers, flanked by conserved N- and C- terminal globular domains, which regulate silk formation (9). The N-terminal (NT) domains from two different silk protein types, major ampullate spidroin 1 (MaSp1) and minor ampullate spidroin (MiSp), have been thoroughly investigated (10, 11, 12, 13, 14, 15, 16, 17). The NT domain of MaSp1—hereafter denoted MaSp NT—from *Euprosthenops australis* contains five-helices and is about 130 amino acid residues long (18, 19, 20). The domain is a highly soluble monomer at a pH above 6.5 (21) and forms an antiparallel homodimer at lower pH (12, 14, 18, 21). On the one hand, when stored in the spider silk gland, the monomeric form of NT promotes solubility of the aggregation-prone repetitive regions at very high spidroin concentrations (up to 50% w/v) (22, 23). On the other hand, during transit through the duct where the pH is gradually lowered, dimerization of NT contributes to fiber assembly by interconnecting spidroins (18). In the dimerization process of MaSp NT, the aspartic acid (Asp) 40 and lysine (Lys) 65 are particularly important. They mediate the initial association of monomer subunits which alters local environments of certain acidic residues and increases their respective pKa. In the dimer, Asp 40 and Lys 65 form an intermolecular salt bridge (24). The transition from monomer to dimer conformation in MaSp NT is mediated by sequential protonation of the three glutamates (Glu) 79, 84, and 119 (21). The tryptophan (Trp) 10 is buried between helix 1 and helix 3 in the monomer and becomes surface exposed during dimerization (18, 19, 20, 25). Relocation of Trp 10 enables the rearrangement of the five helices into a conformation that is compatible with the dimer interface (20).

Two mutants were particularly useful for understanding the dimerization mechanism for *E. australis* MaSp NT. A triple mutant, NT_E79QE84QE119Q_, mimicking the fully protonated state of the three Glu residues described above behaved as a constitutive dimer (21), while a double mutant, named NT∗, in which the residue identities at positions 40 (Asp to Lys) and 65 (Lys to Asp) were interchanged, was prevented from dimerization (26). NT∗ is thermodynamically more stable, has a higher refolding capacity than WT NT, and has been used as a potent solubility tag for recombinant production of aggregation-prone proteins and peptides (26, 27, 28, 29, 30, 31, 32, 33).

NT is the most conserved domain of spidroins (9, 34, 35), and this implies a common mechanism of action across spider species and types of silk. Otikovs *et al.* (12) showed that MiSp NT from *Araneus ventricosus* adopts a mechanism of dimerization comparable to that of MaSp NT. Two Glu residues that become protonated in MaSp NT are conserved in MiSp NT, but Glu 84 is substituted by serine and the nearby Glu 73 (corresponding to Glu 76 in Fig. 1*A*) is instead protonated during MiSp NT dimerization. Similar observations were made in the characterization of MaSp NT from *Latrodectus hesperus*, but the protonating residues could not be identified (14). The nuclear magnetic resonance (NMR) structures of *A. ventricosus* MiSp NT (PDB ID: 2MX8) (12) and *E. australis* MaSp NT (PDB ID: 2LPJ) (20) show that Glu 73 and Glu 84 are located in different parts of the molecule. In MiSp NT, Glu 73 is located in helix 3 close to the center of the dimer interface, whereas Glu 84 in MaSp NT is located in the loop between helix 3 and helix 4. Like MaSp NT, MiSp NT has a Trp that is wedged between helix 1 and 3 in the monomer and changes its fluorescence emission in association with dimerization. Trp 10 is conserved in most of the spidroin NTs, but interestingly, Trp is not present in any of the NTs from flagelliform spidroins (FlSps) (Fig. S1). In FlSp NT, the corresponding residue is replaced by phenylalanine (Phe) or leucine (Leu) except in the species *Steatoda grossa* where it is replaced by a tyrosine (Tyr). The more hydrophobic nature of Phe and Leu, compared to Trp (36, 37), suggests that there is a higher energetic penalty for them to relocate and become exposed to a polar environment during dimerization.

**Figure 1:**
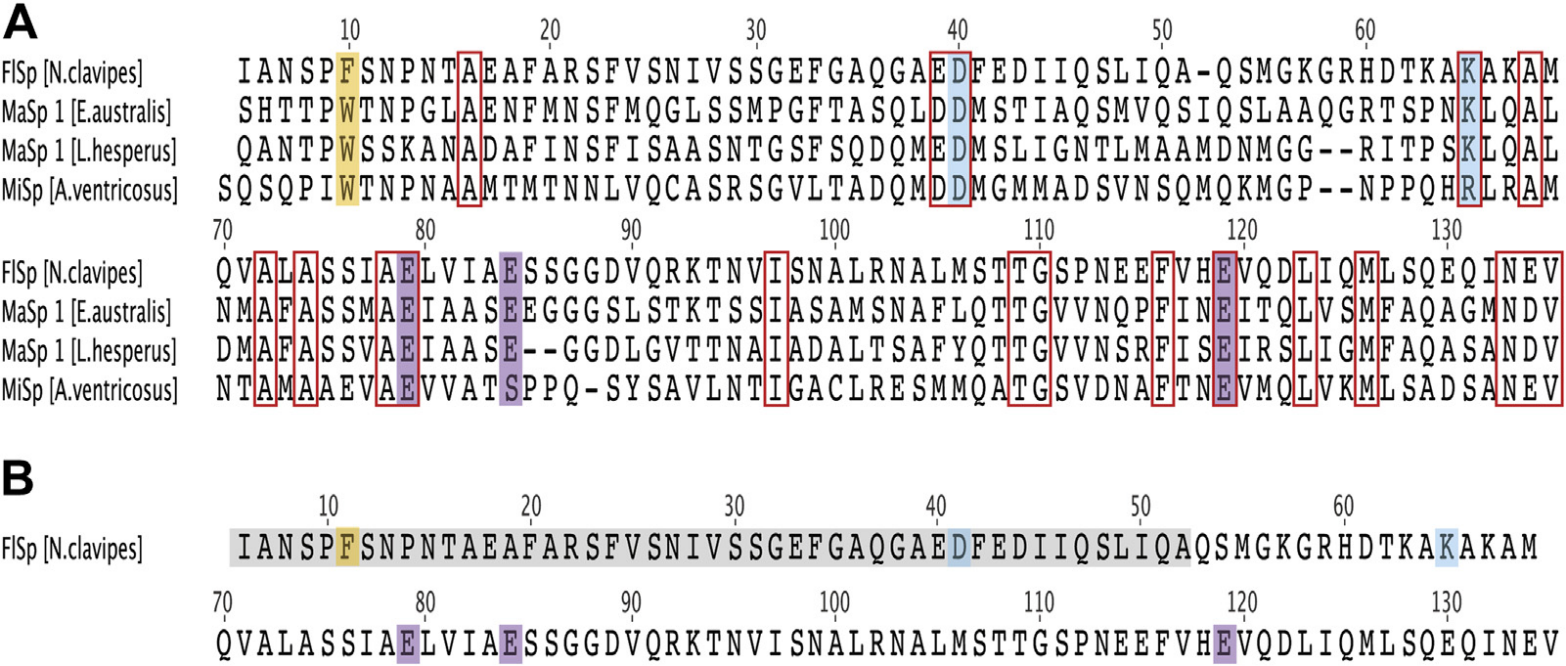
**Comparison of *N. clavipes* FlSp NT with NT domains from other spidroins and spider species.***A*, sequence alignment of FlSp NT from *N. clavipes* with MaSp NT from *E. australis* and *L. hesperus*, and MiSp NT from *A. ventricosus*. Sequence alignment of all known spidroin NTs is shown in Fig. S1. The wedged Trp in MaSp and MiSp NT is substituted by a Phe in FlSp NT (*orange shade*). Positions with strictly conserved residues or conserved side chain charges are *boxed* in *red*. Mutated residues in MaSp NT constitutive monomer and dimer mutants are also indicated in the alignment. Asp 40 and Lys 65 are swapped in NT∗ (*blue shade*). E79, E84, and E119 are mutated to glutamines in NT_E79QE84QE119Q_ (*purple shade*). *B*, the residue numbers used for FlSp NT in this paper differ by +1 at residues 5 to 51 (*gray shade*) compared to MaSp NT and the sequence alignment in (*A*). FlSp, flagelliform spidroin; MaSp, major ampullate spidroin; MiSp, minor ampullate spidroin; NT, N-terminal.

*Nephila clavipes* FlSp NT shares only about 35% residue identity with *E. australis* MaSp NT but still contains all the conserved residues involved in protonation in MaSp NT (Fig. 1*A*), which raises the question whether the dimerization is conserved also in this divergent NT. Herein, we determine the solution NMR and X-ray crystal structures of WT FlSp NT and investigate the dimerization behavior for the WT protein. We also construct FlSp NT mutants that based on MaSp NT data may behave as constitutive monomers or dimers, respectively, as well as Phe 11 to Trp mutants (corresponding to MaSp Trp 10, Fig. 1*B*), and use size-exclusion chromatography (SEC) with or without multiangle light scattering (MALS), electrospray ionization mass spectrometry (ESI-MS), circular dichroism (CD), and NMR spectroscopy to study the effects of these mutations on the structure and ability to form dimers.

## Results

### Structure of *N. clavipes* FlSp NT monomer and dimer

The structures of WT FlSp NT in its monomeric and dimeric conformation were solved by NMR spectroscopy. The polypeptide chain conformation was well defined for most residues, except for the NT tail (residues 1–10) and loop between helices 2 and 3. The X-ray crystal structure of FlSp NT dimer was also solved and contained a single dimer in crystallographic asymmetric unit. The model could be built for most of the protein, excluding NT residues 1 to 9 in subunit A and 1 to 11 in subunit B, C-terminal residue of subunit A and loop, including residues 55 to 59 of subunit A. All the obtained structures were compared to WT MaSp NT in order to determine the overall structural similarity and to identify structural differences for the key residues.

**Figure 2:**
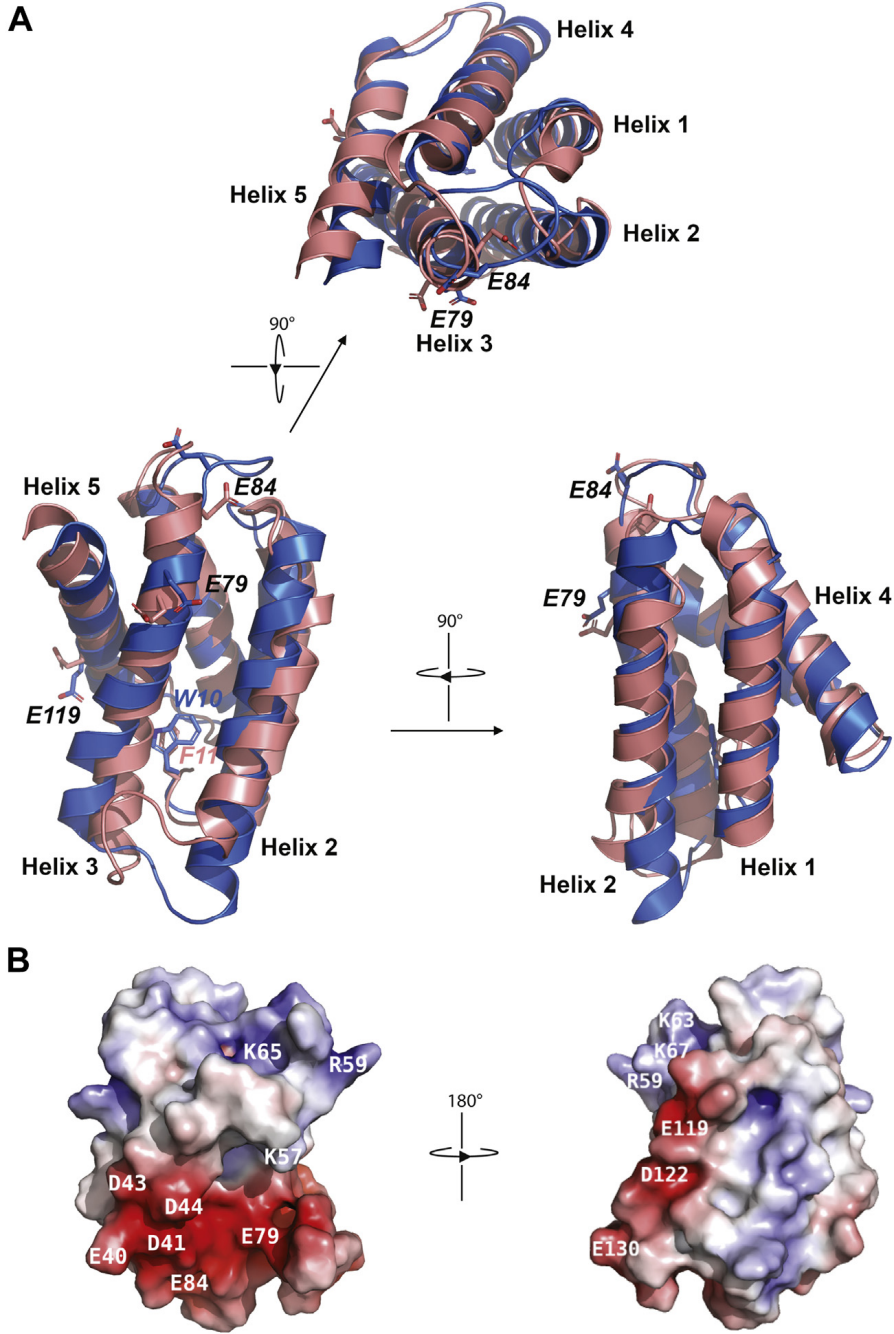
**Comparison of FlSp NT and MaSp NT monomeric structures.***A*, superposition of structures of FlSp NT (*salmon*, PDB ID: 7A0I, this work) and MaSp NT (*blue*, PDB ID: 2LPJ) at pH 7.2. The side chains of Glu 79, Glu 84, Glu 119, and the side chain of Phe 11 in FlSp which corresponds to Trp 10 in MaSp are labeled and displayed as *sticks*. *B*, charge distribution of FlSp NT displayed in a surface representation. The negatively and positively charged residues are colored in *red* and *blue*, respectively. FlSp, flagelliform spidroin; MaSp, major ampullate spidroin; NT, N-terminal.

The structure of the FlSp NT monomer solved at pH 7.2 (PDB ID: 7A0I, this work) shows a high similarity to the structure of monomeric MaSp NT (PDB ID: 2LPJ) as reflected by the RMSD value of 1.84 Å for the polypeptide backbone calculated over 113 residues (Figs. 2*A* and S2*A*). The main difference to MaSp NT is the length of helix 2, which is eight residues shorter in FlSp NT. Additionally, the loop between helix 2 and 3 is three residues longer, and the loop between helix 4 and 5 is three residues shorter in FlSp NT. Phe 11 is wedged between helix 1 and helix 3 in the same orientation as Trp 10 in MaSp NT. As observed in the other characterized NT domains, the charged residues of FlSp NT have a dipolar distribution with clusters of opposite charges at each end (Fig. 2*B*). Arg 59, Lys 57, Lys 63, Lys 65, and Lys 67 cluster at one pole of the protein while Glu 40, Asp 41, Asp 43, Asp 44, Glu 79, and Glu 84 cluster at the other end. However, FlSp NT has a higher number of charged residues (25 against 11 for MaSp NT), and the distribution is not strictly dipolar since charges are also dispersed in the center of the molecule. Two of the conserved residues that become protonated during dimerization of MaSp NT (Glu 79 and Glu 119) show the corresponding localization in FlSp NT.

**Figure 3:**
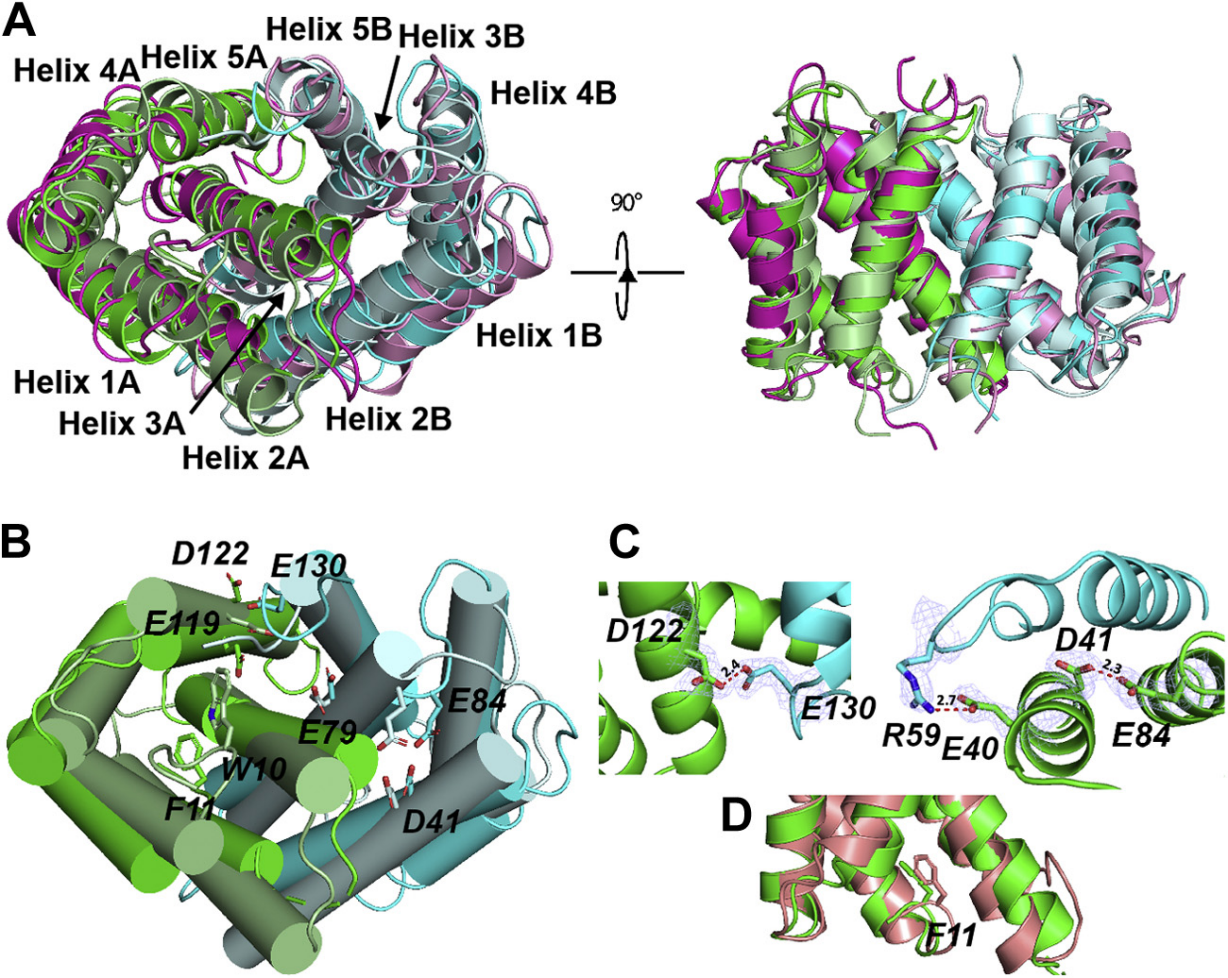
**Comparison of FlSp NT and MaSp NT dimeric structures.***A* and *B*, superposition of structures of FlSp NT (X-ray structure in *green*/*cyan* and NMR in *magenta*/*pink*, PDB IDs: 7OOM and 7A0O, this work) and MaSp NT (*pale green*/*pale cyan*, PDB ID: 3LR2) at pH 5.5. In *B*, the side chains of Asp 41, Glu 79, Glu 84, Glu 119, Asp 122, Glu 130, and the side chain of Phe 11 in FlSp which corresponds to Trp 10 in MaSp are labeled and displayed as sticks. *C*, zoom-in on dimer formation promoting hydrogen bond interactions between protonated acidic residues (Glu 130 and Asp 122 of the other subunit, Glu 84 and Asp 41 of the same subunit, which in turn favors formation of salt bridge among Glu 40 and Arg 59 of the other subunit). Distances are shown in Å between interacting atoms. Electron density contoured at 1σ is shown for interacting residues. *D*, superposition of the NMR structure of FlSp NT monomer at pH 7.2 (*salmon*) and a subunit of FlSp NT dimer X-ray structure (*green*). The side chain of Phe 11 is labeled and displayed as *sticks*. FlSp, flagelliform spidroin; MaSp, major ampullate spidroin; NT, N-terminal.

The structures of the FlSp NT dimer solved by solution NMR and by X-ray crystallography at pH 5.5 (PDB IDs: 7A0O and 7OOM, this work) reveal an antiparallel homodimer similar to the structure of the MaSp NT dimer (PDB ID: 2LTH) as suggested by the RMSD values of 3.8 Å and 3.5 Å, respectively, for the polypeptide backbone calculated over 230 residues (Figs. 3*A* and S2*B*). The corresponding RMSD between the NMR and X-ray structures of FlSp NT dimer is 1.4 Å. The helices have comparable orientations to those in MaSp NT, and the side chains are mostly pointing in the same direction. As observed in MaSp NT, Glu 79 and Glu 119 of FlSp NT are buried at the dimer interface; however, both are involved in salt bridge or hydrogen bonding interactions with Lys 67 and Gln 70, respectively. Therefore, neutralization of these residues upon pH-driven dimerization may not stabilize the dimer by reducing electrostatic repulsion between the subunits. In the NMR structure, Glu 84 is solvent exposed as in MaSp NT, suggesting that it fulfills a similar role in both domains. Glu 130 is facing a cluster of negative charges (Glu 115, Glu 119, and Asp 122) present at the dimer interface, which could lead to the elevation of its pKa value and its subsequent protonation to alleviate electrostatic repulsion during dimer formation. The high-resolution X-ray structure reveals more details on pH-dependent interactions between acidic residues of monomers (Fig. 3*B*). The most obvious such interaction occurs among Asp 122 and Glu 130 of both subunits. This interaction is possible only at low pH and requires that at least one of the acidic residues—presumably Glu 130—is protonated. Another notable contact occurs among Glu 84 and Asp 41 of the same subunit, described previously as “handshake interaction” in MaSp NT (18) (Fig. 3*C*). This interaction in turn may alter the relative placement of α-helices and favor necessary interactions between monomers, such as salt bridge formation among Glu 40 and Arg 59 of the other subunit. However, this salt bridge is present only in one side of the dimer, since the loop, including Arg 59 is disordered in the other monomer of our crystal structure. The intersubunit salt bridge formed between Asp 40 and Lys 65 in MaSp NT is also present between Asp 41 and Lys 65 in FlSp NT and an additional interaction between Asp 44 and Lys 65 was identified. Interestingly, Phe 11 does not swing out upon dimerization, but its side chain is less well defined implying increased dynamics in the dimer (Figs. 3*D* and S2*C*).

### Dimerization studies

The ability of MaSp and MiSp NT to dimerize as a function of pH can be conveniently monitored by Trp fluorescence spectroscopy since Trp 10 undergoes changes in environmental conditions upon dimerization (11, 12, 20, 21, 26). In FlSp NT, the Trp is replaced by Phe 11, and the use of fluorescence spectroscopy to monitor dimerization is prohibited due to the presence of several additional Phe which are not likely to be responsive to dimerization. The dimerization of FlSp NT and its mutants was instead assessed by chromatography and other spectroscopic methods previously used to study MaSp and MiSp NT (12, 14, 15, 17, 20, 21, 26).

**Figure 4:**
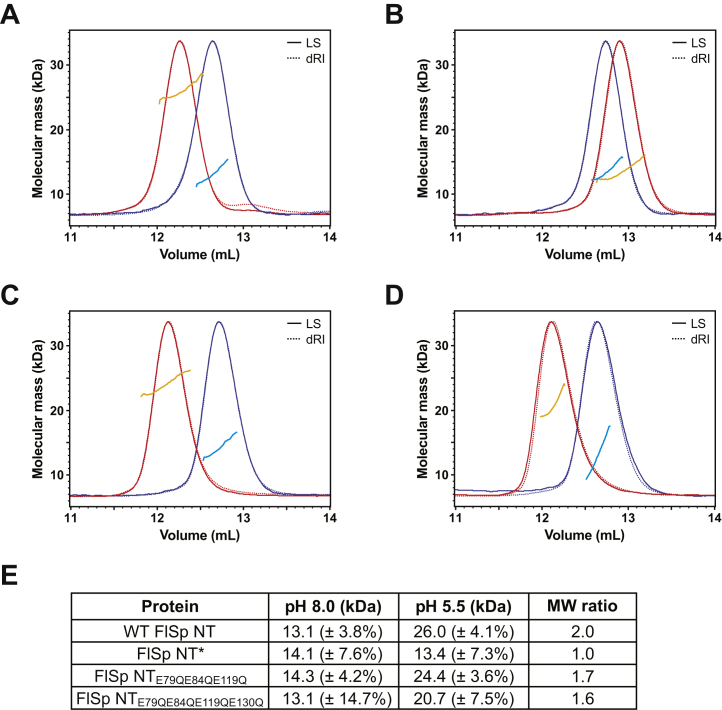
**Dimerization of FlSp NT analyzed by SEC-MALS.** Proteins purified at pH 5.5 and pH 8.0 were analyzed using a Superdex 75 increase SEC column equilibrated at each pH. The chromatograms show the differential refractive index (dRI, *broken lines*) and light scattering signal (LS, *solid lines*) at pH 5.5 (*red*) and pH 8.0 (*blue*) together with the molecular mass calculated by MALS at pH 5.5 (*orange*) and pH 8.0 (*cyan*). Representative measurements are shown for (*A*) WT FlSp NT, (*B*) FlSp NT∗, (*C*) FlSp NT_E79QE84QE119Q_, and (*D*) FlSp NT_E79QE84QE119QE130Q_. MALS data for FlSp NT_E79QE84QE119QE130Q_ were associated with higher uncertainty (%). *E*, the experimentally determined average molecular masses were used to calculate the molecular weight ratio between pH 5.5 and pH 8.0 for each protein. FlSp, flagelliform spidroin; MALS, multiangle light scattering; NT, N-terminal; SEC, size-exclusion chromatography.

SEC was run in the presence of 154 mM NaCl at pH 5.5 and pH 8.0, conditions under which WT MaSp NT shows an apparent molecular weight (MW) ratio of 1.4 between dimer and monomer when comparing the elution volumes with a set of calibrants (26). In this experimental setup, WT FlSp NT (calculated mass of monomer 14.2 kDa) shows an MW of 27.4 kDa at pH 5.5 and 21.5 kDa at pH 8.0 with an apparent MW ratio of 1.3 between dimer and monomer (Fig. S3). Due to the less distinguishable difference in elution volumes for WT FlSp NT monomer and dimer, we chose to determine the MWs more accurately by SEC-MALS. This resulted in MW of 13.1 kDa ± 3.8% at pH 8.0 and 26.0 kDa ± 4.1% at pH 5.5, which gives an MW ratio of 2.0 between dimer and monomer (Fig. 4, *A* and *E*). Further in agreement with the results for MaSp NT, the constitutive monomer mutant FlSp NT∗ remains monomeric independently of pH as its elution volume and MW does not shift significantly between pHs (Fig. 4, *B* and *E*), indicating that the electrostatics around Asp 41 and Lys 65 are crucial also for FlSp NT dimerization. However, FlSp NT_E79QE84QE119Q_ does not behave like a constitutive dimer but keeps its responsiveness to pH, with an MW ratio of 1.7 between pH 5.5 and 8.0 (Fig. 4, *C* and *E*). The herein identified interaction between Asp122 and Glu 130 in the X-ray structure led us to also exchange Glu 130 for Gln in the quadruple mutant FlSp NT_E79QE84QE119QE130Q_. No significant change in pH responsiveness was observed compared to NT_E79QE84QE119Q_ (Fig. 4, *D* and *E*).

**Figure 5:**
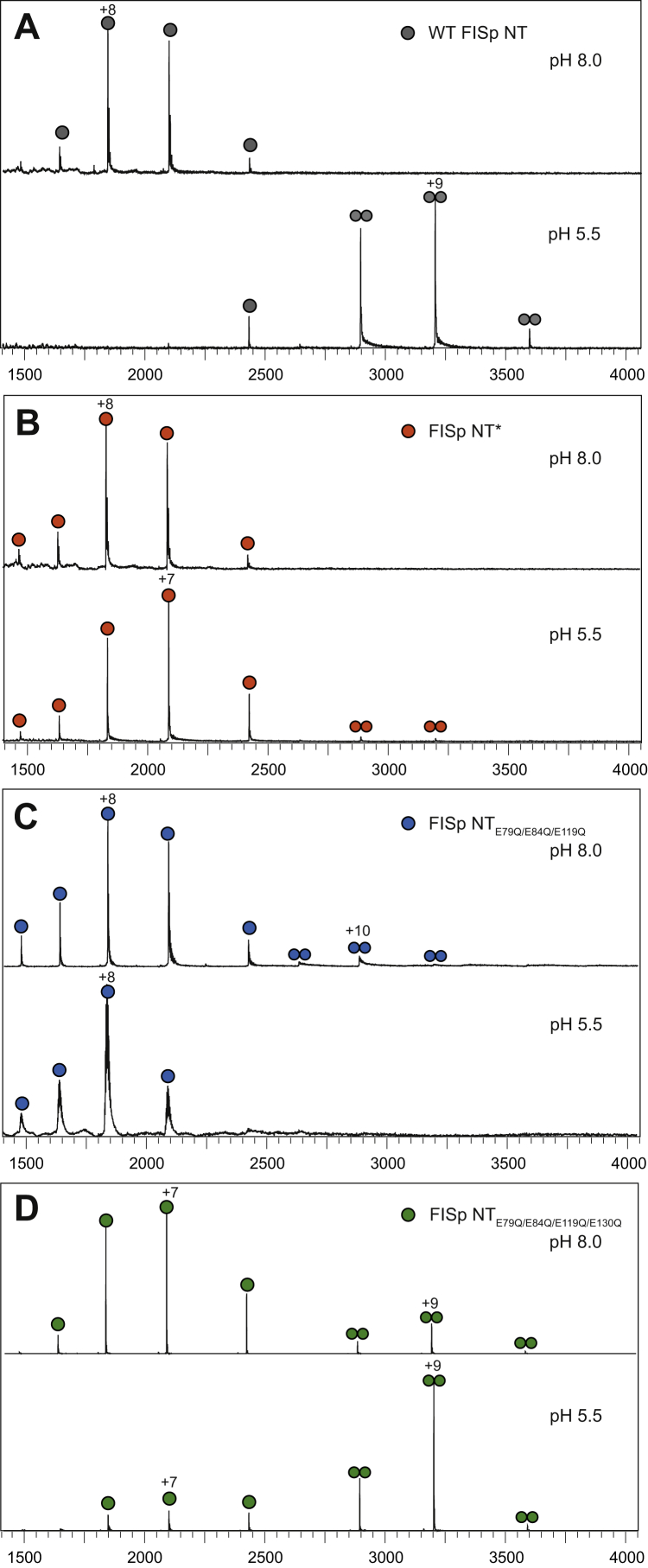
**Dimerization of FlSp NT analyzed by ESI-MS.** Spectra were measured at pH 8.0 and 5.5 of (*A*) WT FlSp NT, (*B*) FlSp NT∗, (*C*) FlSp NT_E79QE84QE119Q_, and (*D*) FlSp NT_E79QE84QE119QE130Q_. ESI-MS, electrospray ionization mass spectrometry; FlSp, flagelliform spidroin; NT, N-terminal.

In accordance with these results, ESI-MS of WT FlSp NT shows monomers at pH 8.0 and dimers at pH 5.5 (Fig. 5*A*) in a concentration-independent manner between 7 and 125 μM (Fig. S4). FlSp NT∗ remains monomeric at both pHs (Fig. 5*B*), while FlSp NT_E79QE84QE119QE130Q_ populates both states, with a higher population of monomers at pH 8.0 and a higher population of dimers at pH 5.5 (Fig. 5*D*). The analysis of FlSp NT_E79QE84QE119Q_ using ESI-MS contradicts the observation made from SEC-MALS as the protein behaves mainly as monomers at both pHs (Fig. 5*C*). FlSp NT_E79QE84QE119QE130Q_ and FlSp NT_E79QE84QE119Q_ in particular most likely form weaker dimers compared to WT FlSp NT due to neutralization of residues not involved in the dimerization process. Hence, experimental differences such as lower stability during evaporation, lower concentration, and a different buffer composition could explain the discrepancy between ESI-MS and SEC-MALS data.

**Figure 6:**
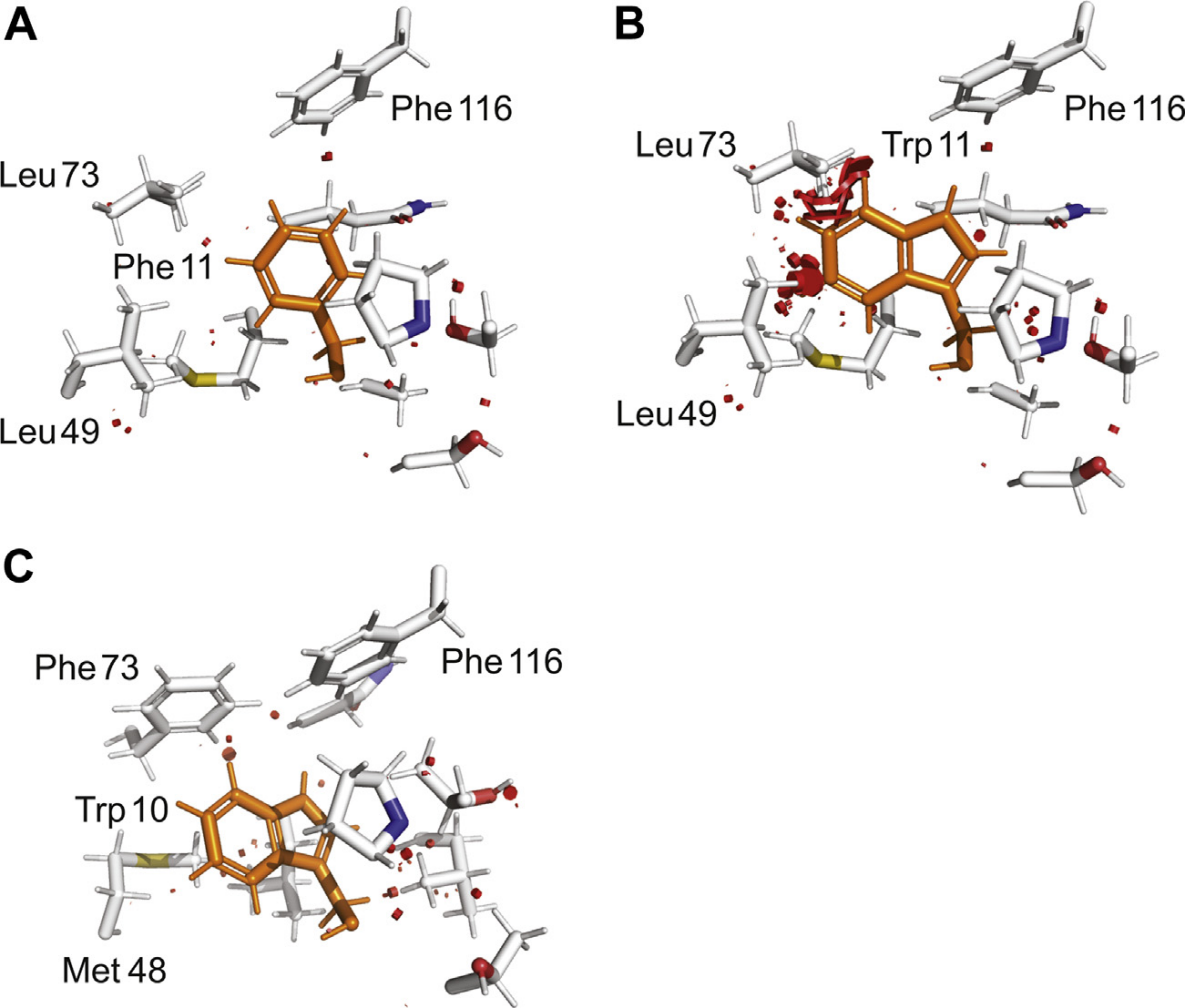
**Close-up views of Trp/Phe pockets.** Side chains surrounding (*A*) Phe 11 in FlSp NT, (*B*) modeled Trp 11 in FlSp NT_Trp_, and (*C*) Trp 10 in MaSp NT. The main unfavorable contacts are highlighted in *red*. FlSp, flagelliform spidroin; MaSp, major ampullate spidroin; NT, N-terminal.

To evaluate the importance of Phe/Trp 11 in the dimerization process, we designed Phe to Trp mutants of FlSp NT and NT∗ (referred to as NT_Trp_ and NT∗_Trp_). The pocket accommodating Phe 11 is too shallow for a Trp side chain to fit in, due to steric hindrance from the side chains of Leu 49 and Leu 73 (Fig. 6, *A* and *B*). This suggests that Trp 11 of NT_Trp_ either is surface exposed or that rearrangements of the surrounding side chains and/or helices allow it to be accommodated. In comparison, the positioning of Trp 10 between the helices in MaSp NT is allowed by the orientation of the side chains of Met 48 and Phe 73 (Fig. 6*C*) and a less compact arrangement of helix 2 and 3 in relation to the rest of the structure compared to FlSp NT (Fig. 2*A*). SEC analysis of FlSp NT_Trp_ showed an apparent MW ratio of about 1.2 between pH 5.5 and 8.0, whereas NT∗_Trp_ remained monomeric at both pH (Fig. S5*A*), showing that the Phe to Trp mutation has no influence on the dimerization behavior of FlSp NT, or the lack thereof for FlSp NT∗. No Trp fluorescence emission maximum shift was however observed upon lowering the pH (Fig. S5, *B* and *C*), which indicates that Trp in NT_Trp_ does not change its location during dimerization in a way that influences fluorescence emission maximum. However, the decrease of pH led to a decrease in fluorescence intensity for NT_Trp_ but not for NT∗_Trp_ (Fig. S5, *B* and *C*), likely due to quenching of the Trp fluorescence by the protonation of neighboring residues during dimerization.

### NMR studies of conformations at pH 5.5 and 7.2

**Figure 7:**
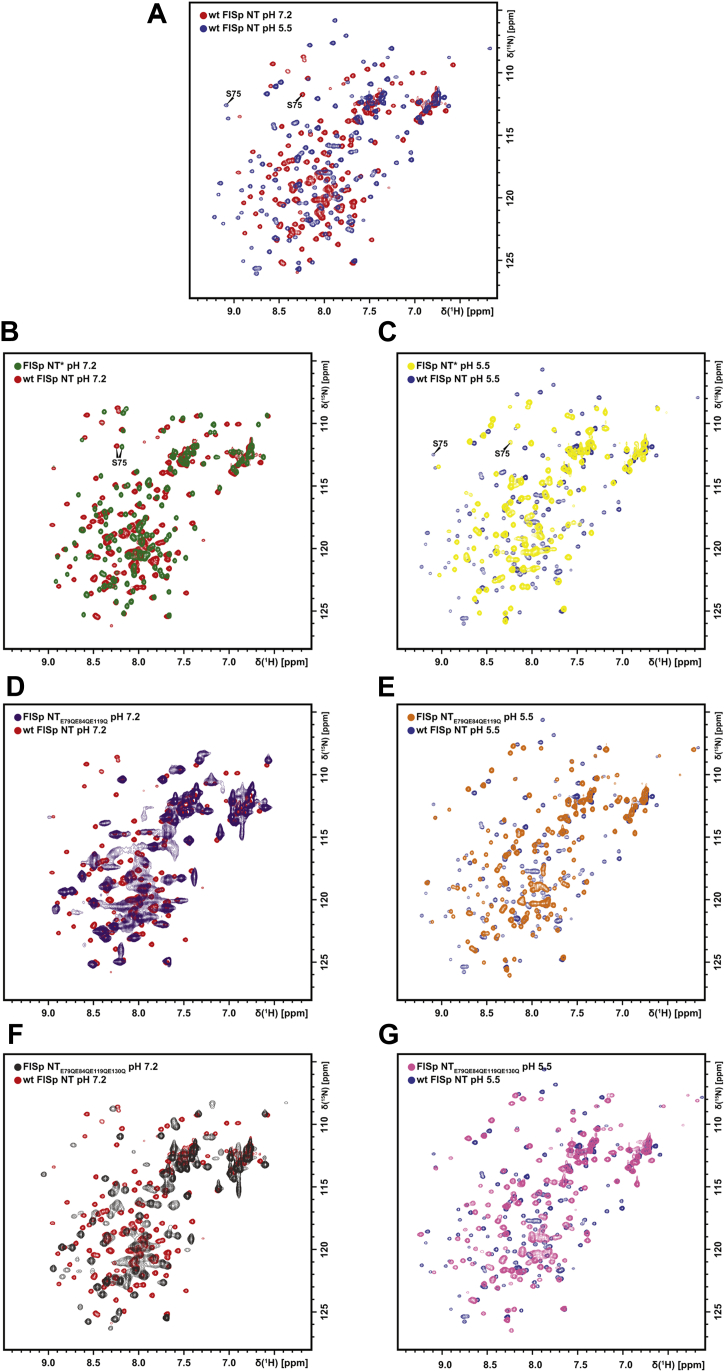
**HSQC NMR spectra of WT FlSp NT and mutants.** Overlaid spectra of (*A*) WT FlSp NT at pH 7.2 (*red*) and pH 5.5 (*blue*), (*B*) FlSp NT∗ (*green*), and WT FlSp NT (*red*) at pH 7.2, (*C*) FlSp NT∗ (*yellow*) and WT FlSp NT (*blue*) at pH 5.5, (*D*) FlSp NT_E79QE84QE119Q_ (*purple*) and WT FlSp NT (*red*) at pH 7.2, (*E*) FlSp NT_E79QE84QE119Q_ (*orange*) and WT FlSp NT (*blue*) at pH 5.5, (*F*) FlSp NT_E79QE84QE119QE130Q_ (*gray*) and WT FlSp NT (*red*) at pH 7.2, (*G*) FlSp NT_E79QE84QE119QE130Q_ (*pink*) and WT FlSp NT (*blue*) at pH 5.5. FlSp, flagelliform spidroin; HSQC, heteronuclear single quantum coherence; NT, N-terminal.

Heteronuclear single quantum coherence (HSQC) NMR spectra of WT FlSp NT and the mutants were recorded at pH 5.5 and pH 7.2 (Figs. 7 and S6). Comparison of the spectra of WT FlSp NT at both pH (Fig. 7*A*) confirms that the domain changes conformation as highlighted by the large chemical shift differences recorded upon lowering the pH (Fig. S6*A*). Molecular self-diffusion coefficients (D_s_) were 12.3 × 10^−7^ cm^2^/s at pH 7.2 and 9.7 × 10^−7^ cm^2^/s at pH 5.5 (Fig. S7) as measured by pulsed-field gradient (PFG)-NMR. These values are in good agreement with the theoretically estimated ratio of 0.75 for D_s,dimer_/D_s,monomer_ according to the Stokes–Einstein equation (38). Furthermore, ^15^N NMR relaxation analysis of WT FlSp NT revealed rotational correlation times consistent with a MW of 16.8 kDa at high pH and 26.4 kDa at low pH (Fig. S6*E*), further confirming that FlSp NT dimerizes as observed by SEC-MALS and ESI-MS.

Overlay of the spectra of WT FlSp NT and FlSp NT∗ revealed comparable spectra at pH 7.2 but dissimilar spectra at pH 5.5, confirming that FlSp NT∗ is monomeric at both pH (Fig. 7, *B* and *C*). The residue Ser 75 located in the middle of the dimeric interface can serve as a sentinel residue for the dimer and monomer conformations. Its H^N^ chemical shift changes by almost 1 ppm between pH 8.0 and 5.5 for WT FlSp NT, whereas for FlSp NT∗, the Ser 75 H^N^ chemical shift remains almost unchanged. The slight differences between FlSp NT∗ spectra upon lowering the pH (Fig. S6*B*) might be due to a more expanded monomeric conformation at low pH compared to high pH, as observed by relaxation experiments (Fig. S6*E*) or due to nonspecific interaction with the buffer components.

The NMR data show that FlSp NT_E79QE84QE119Q_ and NT_E79QE84QE119QE130Q_ retain responsiveness to decrease of pH as indicated by a change of the spectrum from pH 7.2 to 5.5 (Fig. S6, *C* and *D*). At pH 7.2, the spectra are more similar to the spectrum of WT NT (Fig. 7, *D* and *F*) but with several broadened peaks, indicating that the mutant domains are in equilibrium between a higher-populated monomeric conformation and less-populated dimeric conformation. However, at pH 5.5, the spectra display more peaks than at higher pH (Fig. S6, *C* and *D*) but less peaks compared to WT NT at the same pH (Fig. 7, *E* and *G*). This could indicate that FlSp NT_E79QE84QE119Q_ and NT_E79QE84QE119Q130Q_ adopt a less stable and/or more dynamic dimer at pH 5.5. Relaxation experiments suggest that FlSp NT_E79QE84QE119Q_ and NT_E79QE84QE119QE130Q_ are dimers as the recorded molecular tumbling rate is more consistent with the size of a dimer at both pH (Fig. S6*E*), although the apparent τ_c_ values at pH 7.2 are likely increased due to the observed exchange process that leads to signal broadening.

### Refolding capacity and thermal stability

The different FlSp NT variants were analyzed by CD spectroscopy to evaluate the refolding capacity at pH 8.0 and pH 5.5 (Fig. S8*A*). Spectra were recorded at 25 °C, after heat denaturation at 95 °C and after lowering the temperature back to 25 °C. At both pH, WT FlSp NT as well as FlSp NT∗ and NT_E79QE84QE119Q_ adopt mainly α-helical secondary structures at 25 °C, as evidenced by double minima at 208 and 222 nm. The structures convert to random-coil with a broad minimum around 204 nm upon heat denaturation and refold to almost identical α-helical features as before heating when lowering the temperature back to 25 °C. The thermal stability of each protein was assessed by heat-induced denaturation, and the melting temperatures (T_m_) were determined from the half-way transition points between the folded and unfolded states (Fig. S8*B*). At pH 8.0, the thermal stability of WT FlSp NT (62 °C) is higher than the T_m_ reported for WT MaSp NT (54 °C) (21). Both FlSp NT∗ and FlSp NT_E79QE84QE119Q_ are more stable than WT FlSp NT at this pH, showing T_m_ of 69 °C and 73 °C, respectively. The increased stability of FlSp NT∗ and NT_E79QE84QE119Q_ may be caused by the removal of a destabilizing effect from close proximity of Asp 41 and Glu 79 side chains across a helical interface. In NT∗, a new salt bridge between Lys 41 and Glu 79 could stabilize the protein fold, while NT_E79QE84QE119Q_ could be stabilized by abolishing charge repulsion as well as by facilitating subunit association into a dimer-like conformation. At pH 5.5, all variants have an increased stability with WT FlSp NT showing the highest T_m_ (82 °C).

## Discussion

We characterized WT FlSp NT from *N. clavipes* by solution NMR spectroscopy and X-ray crystallography and showed that this domain adopts a five-helix arrangement (Figs. 2 and 3) which is overall very similar to the structure of monomeric and dimeric MaSp NT. The pH-dependent dimerization is conserved also in FlSp NT as shown by SEC-MALS (Fig. 4*A*), ESI-MS (Fig. 5*A*), HSQC- and PFG-NMR spectroscopy (Figs. 7*A*, S6 and S7). In order to examine the underlying molecular mechanism of the dimerization of FlSp NT, we designed FlSp NT∗ and FlSp NT_E79QE84QE119Q_, corresponding to two MaSp NT mutants that are independent of pH and mimic the monomeric and dimeric states, respectively (21, 26). In addition, Glu 130 was mutated in FlSp NT_E79QE84QE119QE130Q_ to investigate its potential role in the dimerization. We found evidence that FlSp NT∗ indeed behaves as a constitutive monomer insensitive to pH (Figs. 4*B*, 5*B* and 7, *B* and *C*). Interestingly, the analyses of FlSp NT_E79QE84QE119Q_ and NT_E79QE84QE119QE130Q_ were less conclusive, and partly contradictory results were obtained. ESI-MS suggested that NT_E79QE84QE119Q_ is unable to dimerize (Fig. 5*C*) while NT_E79QE84QE119QE130Q_ contains both monomeric and dimeric populations at both pHs (Fig. 5*D*). SEC, SEC-MALS, and NMR spectroscopy pointed either toward a pH-sensitive dimerization (Fig. 4, *C* and *D*) or a significant population of an intermediate conformation (Figs. 7, *D*–*G* and S6, *C* and *D*).

Electrostatic interactions assisted by Asp 40 and Lys 65 of two NT subunits have been proposed to be crucial for the association of MaSp NT monomers into dimers (21). Swapping of these charges should theoretically still allow formation of a salt bridge, but the rearrangement of charges may have a detrimental effect on charge clusters that normally give elevated pKa values for acidic residues and enable them to become protonated in the dimer. Indeed, exchanging the position of the corresponding residues Asp 41 and Lys 65 in FlSp NT∗ resulted in the abolition of the dimerization process, in excellent agreement with the behavior for MaSp NT∗. The latter is a constitutive monomer which has been used in biotechnological applications as a highly efficient solubility enhancer for the production of aggregation-prone proteins (26, 27, 28, 30, 31, 32, 33), and it has recently been shown that FlSp NT∗ outperforms MaSp NT∗ as a solubility enhancer for the very aggregation-prone amyloid beta 42 peptide (29). The higher capacity of FlSp NT∗ to promote solubility might lie in the greater number of charged residues and the higher absolute net charge (25 charges, −7 of net charge) compared to MaSp NT (11 charges, −5 of net charge). However, the basis for solubilization is not fully understood and likely is an outcome from the combined properties of the tag and the protein of interest. Nature provides an array of NTs from different spider species which likely are dedicated for solubilizing a particular silk type and show different degrees of sequence divergence. This also offers a future opportunity to screen for novel tags with specialized features.

In MaSp NT from *E. australis*, Glu 79, Glu 84, and Glu 119 were identified as the three residues that mediate the NT dimerization *via* a three-step protonation mechanism (21). Moreover, Glu 79 and Glu 119 were shown to play similar roles in MiSp NT from *A. ventricosus* and in MaSp NT from *L. hesperus* although the role of Glu 84 was taken over by Glu 73 and a residue that has yet to be identified, respectively (12, 14). Glu 79, Glu 84, and Glu 119 are well conserved in NT domains among the different types of silk and different spider species (Fig. S1). Interestingly, all NTs have either Glu or Asp in position 79. We hypothesized that strict conservation reflects a key role of residue 79 in the dimerization process, which likely applies to FlSp NT as well. Glu 84 has a similar orientation in FlSp and MaSp NT, indicating that it may have the same role in both domains. In MaSp NT, Glu 84 requires the protonation of Glu 79 and Glu 119 before capturing a proton. However, our investigation shows that the residues involved in FlSp NT dimerization do not directly correspond to the three Glu residues identified in MaSp NT. Failing to identify the second residue to be protonated in FlSp NT might explain why FlSp NT_E79QE84QE119Q_ is unable to form a constitutive dimer. Glu 119 may have merely a structural role in FlSp NT as the structure now determined for the FlSp NT dimer shows that the side chain of Glu 119 could form a hydrogen bond with Gln 70 and consequently bring helix 3 closer to helix 5. MaSp NT has an asparagine (Asn) at position 70, but since the Asn side chain is one methylene group shorter than that of Gln, the formation of a corresponding hydrogen bond is not seen in MaSp NT. Acidic residues nearby Glu 119 in FlSp NT, *i.e.*, Glu 115, Asp 122, and Glu 130 are lacking counterparts in MaSp and MiSp, and one or several of them could potentially replace Glu 119 as titrating residue(s) during FlSp NT dimerization. Based on the X-ray structure of FlSp NT, Glu 130 was identified as a promising candidate as its protonation would favor dimer formation by alleviating the electrostatic repulsions with negatively charged residues present at the dimer interface, and this supposition agrees with major chemical shift changes of Gln 131 upon dimerization of FlSp NT (Fig. S6*A*). When introducing the Glu 130 to Gln mutation in the FlSp NT_E79QE84QE119QE130Q_ mutant, there was a shift toward a dimer population compared to NT_E79QE84QE119Q_ when analyzed with ESI-MS (Fig. 5, *C* and *D*) but neither SEC-MALS or HSQC NMR showed any significant difference in the pH responsiveness between the two mutants. However, a set of combined mutations that do not reflect the exact protonation pattern in the WT dimer may have detrimental effects on the charge clusters, and one should not rule out that mutation of Glu 130, and/or any of the other investigated residues, may promote dimer formation if combined differently or together with a yet not identified residue.

The characterization of NT_Trp_ and NT∗_Trp_ showed that replacing Phe by Trp at position 11 has no negative influence on the ability of FlSp NT to dimerize (Fig. S5*A*). However, the Trp does not relocate upon dimerization (Fig. S5, *B* and *C*), suggesting that the different conformations of Trp 10 as observed in the MaSp dimer and monomer are merely a consequence of dimerization and not a requirement for the structural conversion. In comparison to the structure of FlSp NT, helix 2 and 3 are further away from the rest of the molecule in MaSp NT, which might allow Trp 10 to swing out from a buried to a solvent exposed position upon dimerization. Still, it is possible that Trp in NT_trp_ changes its position between monomer and dimer in a way that does not result in enough change of the surrounding polarity to influence its fluorescence properties. The pocket for accommodating Phe in monomeric FlSp NT is smaller than the pocket for Trp in MaSp NT, and as a consequence, the side chain of Trp in NT_Trp_ is too bulky to entirely fit in this pocket (Fig. 6) and hence may become partly solvent-exposed already in the monomer. Further structural studies are required to explain the importance of Trp and Phe for the monomer to dimer conversion of NT.

Altogether, the presented results show that FlSp NT undergoes pH-dependent dimerization in a process driven by electrostatic interactions and protonation of key residues. Furthermore, the results indicate that the exposure of Trp 10 in the MaSp NT dimer is incidental to the rearrangement of helices during dimerization. We conclude that the Asp 41–Lys 65 pair plays a key role in the initiation of the dimerization as for MaSp NT, while the protonation pattern is different. One or more of the mutated glutamates in FlSp NT_E79QE84QE119QE130Q_ do not become protonated in the FlSp NT dimer, and additional, yet unidentified, carboxylate(s) likely are involved in the protonation process. This is a rather unexpected finding since the three protonating glutamates in MaSp NT are highly conserved and have been maintained in FlSp NT despite the low overall sequence identity. What would be the driving force to evolve a different protonation pattern in a protein with such similar structure and mechanism of action? It is possible that this is a consequence of diverse chemical environments in different types of glands. For example, the short spinning duct of the flagelliform gland theoretically implies a steeper or more narrow pH gradient compared to the major ampullate gland. Changes in the quantity and distribution of nonconserved charged residues will indirectly modify the overall pKa of dimerization, since the pKa for each carboxylate is determined by the local environment and the proximity to charge clusters. An interesting question is whether variations in pKa of dimerization impact the affinity of the NT dimers and the speed of silk formation when the proteins are subjected to the pH gradient along the spinning duct. Further studies are warranted to understand this relationship and how it could impact the structural and mechanical properties of different silk types.

## Experimental procedures

### Subcloning of WT FlSp NT

The gene for WT FlSp NT (GenScript) was amplified by PCR using HiFi HotStart DNA polymerase (Kapa Biosystems) at 60 °C annealing temperature, with the sense primer 5′-TATATTGAATTCAGCTTCACAGTCGCCATTTAGC-3′ and the antisense primer 5′-ATATATAAGCTTACACTTCATTAATCTGTTCCTGCGA-3′. The PCR product was cleaved with restriction enzymes *EcoRI* and *HindIII* (Thermo Scientific) and ligated into the vector pT7His-Trx-His (21) that was previously cleaved with the same enzymes. The new construct was transformed to chemically competent *Euprosthenops coli* Nova Blue cells by heat shock. Plasmids were prepared using QIAprep Spin Miniprep Kit (QIAGEN) according to the manufacturer’s recommendations and were sequenced to verify the identity of the inserted gene.

### Site-directed mutagenesis of FlSp NT mutants

The created vector pT7His-Trx-His-NT_FlSp_ was subjected to point mutagenesis using the QuickChange site-directed mutagenesis kit (Agilent Technologies) according to the manufacturer’s recommendations. The potential constitutive monomer mutant NT∗ (NT_D40KK65D_) and the potential constitutive dimer mutants NT_E79QE84QE119Q_ and NT_E79QE84QE119QE130Q_ were made by successive point mutagenesis, while the Phe to Trp FlSp NT mutants NT_Trp_ and NT∗_Trp_ were made by a single point mutagenesis. The plasmids were transformed into chemically competent *E. coli* Nova Blue cells by heat shock and were prepared as above and subjected to sequencing to verify the sequences of the mutated genes.

### Protein expression and purification

The plasmids were used to transform chemically competent *E. coli* BL21 (DE3) cells. Overnight cultures were inoculated 1/100 to LB medium containing kanamycin (70 mg/l) and grown at 30 °C to an *A*_600_ of 0.9. The cultures were then induced by addition of IPTG to a final concentration of 0.5 mM and were further incubated overnight at 20 °C. The cells were harvested by centrifugation at 5000*g* for 20 min, resuspended to 30 ml in loading buffer (20 mM Tris-HCl pH 8.0), and stored at −20 °C overnight. After thawing, the cells were lysed in a cell disrupter (Constant Systems Ltd) at 30 kPsi. The lysates were cleared by centrifugation at 24,000*g* for 30 min. Analysis of the cleared lysates and the remaining pellets was performed by SDS-PAGE using 4 to 20% Mini-PROTEAN TGX polyacrylamide gels (Bio-Rad Laboratories, Inc), stained with Coomassie brilliant blue dye.

Each supernatant after lysis was loaded onto an immobilized-metal affinity chromatography column packed with Ni-Sepharose (GE Healthcare) and equilibrated with loading buffer. The column was washed with 10 mM imidazole, 20 mM Tris-HCl, pH 8.0 and bound protein was eluted in 2 ml fractions using 300 mM imidazole, 20 mM Tris-HCl, pH 8.0. The absorbance at 280 nm was measured for each fraction, and protein-containing fractions were pooled. Dialysis was performed using a Spectra/Por dialysis membrane (6–8 kDa MW cut-off, Spectrum Laboratories) incubated overnight in 5 l of loading buffer. The fusion protein was cleaved with thrombin (Merck) during dialysis by a thrombin:fusion protein ratio (w/w) of 1:3000 in order to release the Trx tag. After dialysis, the sample was loaded to an immobilized-metal affinity chromatography column previously equilibrated with loading buffer and the unbound pure NT variant was collected. The protein concentration was determined using the Pierce bicinchoninic acid protein assay kit (Thermo Fisher Scientific) according to the manufacturer’s recommendations using a known concentration of MaSp NT∗ as standard. The correct size of the protein was confirmed by SDS-PAGE using a 4 to 20% Mini-PROTEAN TGX polyacrylamide gel (Bio-Rad Laboratories, Inc), stained with Coomassie brilliant blue dye.

For preparation of samples for NMR spectroscopy, an overnight culture was used for a 1/100 inoculation to 500 ml minimal medium M9, supplemented with 70 mg/l kanamycin, containing ^13^C-labeled glucose and ^15^N-labeled ammonium chloride for the expression of WT FlSp NT and only ^15^N-labeled ammonium chloride for the expression of FlSp NT∗, FlSp NT_E79QE84QE119Q_, and FlSp NT_E79QE84QE119QE130Q_. Proteins were expressed and purified as described above and were concentrated to about 1 mM at 4000*g* in a VivaSpin 20 concentrator tube with a 10 kDa MW cut-off (GE Healthcare). Gel filtration on a PD-10 column (GE Healthcare) was performed to exchange the buffer for 20 mM sodium phosphate, 300 mM NaCl at pH 7.2. The samples were concentrated again to about 1 mM and for studies at pH 5.5 exchanged into 20 mM sodium acetate-d_3_ buffer, pH 5.5, 20 mM NaCl, as described above.

Se-Met labeled protein was produced in *E. coli* B834 (DE3). The cells were grown in modified 2xTYP media, supplemented with 133 mM phosphate buffer (pH 7.4) and glucose (2 g/l) until *A*_600_ of 1.0 was reached. The cells were then centrifuged, and the resulting pellet was resuspended in 0501 media without methionine (Athena Enzyme Systems), supplemented with 0502 media (Athena Enzyme Systems) and glucose (5 g/l). Cells were grown for an additional 2 h. IPTG was added to 0.5 mM, mixture of Se-Met:Met (5:1) was added to 0.2 mM, and the cultivation was continued for 20 h. Purification of Se-Met–labeled protein was performed in the same way as for native protein.

### Size-exclusion chromatography

SEC on WT FlSp NT, FlSp NT_Trp_, and FlSp NT∗_Trp_ was performed on an ÄKTA Pure system using a flow rate of 0.7 ml/min. Samples (200 μl) of 2 mg/ml protein solutions were injected, and the column was equilibrated with the respective buffers (20 mM Tris-HCl, 154 mM NaCl, and 1 mM EDTA at pH 8.0 or 20 mM MES, 154 mM NaCl, and 1 mM EDTA at pH 5.5) before each run. The proteins were analyzed on a Superdex 75 Increase 10/300 GL column (GE Healthcare) with UV detection at 280 nm for NT_Trp_ and NT∗_Trp_ or at 215 nm for all other variants. A molecular mass standard set consisting of ribonuclease (13 kDa), carbonic anhydrase (29 kDa), ovalbumin (44 kDa), and conalbumin (75 kDa) was chromatographed at both pH 8.0 and 5.5 to estimate the apparent MWs.

### SEC-MALS

Prior to SEC-MALS experiments, proteins WT FlSp NT, FlSp NT∗, FlSp NT_E79QE84QE119Q_, and FlSp NT_E79QE84QE119QE130Q_ were thawed at 4 °C and visually inspected for precipitation. Subsequently, the samples were spun down at 25,830*g*, 20 min at 4 °C. The volume corresponding to 50 μg of material was loaded onto a Superdex 75 Increase 10/300 GL column (Cytiva) using an Agilent 1260 Infinity Standard Autosampler. All samples were run in duplicate, and 1 mg/ml BSA was used as reference during the runs. The mobile phase flow was provided by an Agilent 1260 Infinity Quaternary Pump, set to a flow rate of 1 ml/min. The column was equilibrated in 20 mM Tris-HCl, 150 mM NaCl, and 1 mM EDTA, pH 8.0 or 20 mM MES, 150 mM NaCl, and 1 mM EDTA, pH 5.5. The buffers were filtered with a 0.1 μm membrane before use. All the experiments were performed at room temperature. The column was connected to a downstream Multiangle Light Scattering Detector (miniDAWN TREOS, Wyatt, equipped with three MALS Detectors at the following angles: 43.63°, 90°, and 136.37°) and to a differential Refractive Index Detector (Optilab T-Rex, Wyatt). The proteins molar masses were then calculated with ASTRA 7.3.2.19, using a dn/dc of 0.185 ml/mg. The light scattering and refractive index signals were corrected using the autobaseline finder function and subsequently an area of the protein peak, ranging from 50 to 60% of the total peak area, was selected and used for the calculations. The proteins molecular mass was calculated using the Zimm method (39). The values of proteins concentration used when fitting the Zimm equation were derived from the RI signal.

### Electrospray ionization mass spectrometry

The proteins were buffer exchanged into 100 mM ammonium acetate, pH 5.5 or pH 8.0, using Biospin six microcentrifuge columns (Bio-Rad). Regarding concentration gradient experiment on WT FlSp NT, 2 mM protein stock was buffer exchanged into 100 mM ammonium acetate, pH 5.5, followed by bicinchoninic acid assay to determine the concentration using a known concentration of MaSp NT∗ as standard. The sample was further diluted to obtain the following concentrations: 7, 15, 31, 62, and 125 μM. Spectra were acquired on a Micromass LCT ToF (MS Vision) equipped with an offline nanospray source. Samples were introduced *via* coated borosilicate capillaries (Thermo Scientific). The voltage of the capillary, the radio frequency lens, and the sample cone was 2.0 kV, 1.5 kV, and 100 V, respectively. The mass scale was calibrated using cesium iodide. Data were analyzed using the MassLynx 4.1 software.

### Tryptophan fluorescence spectroscopy

Tryptophan fluorescence measurements were taken at pH values ranging from 5.2 to 8.0 in 0.4 pH unit steps with a Tecan safire^2^ spectrofluorometer using Costar black polystyrene assay plates (96 flat-bottom wells). The proteins were diluted to a concentration of 10 μM in 20 mM Hepes/20 mM MES buffer. The samples were excited at 280 nm (bandwidth 5 nm), and emission spectra were taken from 300 nm to 400 nm (bandwidth 10 nm) with 1 nm steps.

### NMR spectroscopy

For the structural analysis of WT FlSp NT in solution, NMR spectroscopy experiments were performed at 298 K on a 600 MHz Bruker Avance Neo spectrometer equipped with a 5-mm ^1^H/^19^F (22) quadruple resonance inverse (QCI) cryo-probe. The spectra for 1.2 mM uniformly ^13^C, ^15^N-labeled monomeric WT FlSp NT were recorded in 20 mM sodium phosphate, pH 7.2, 300 mM NaCl, 0.03% (w/v) NaN_3_, 10% D_2_O (v/v). The spectra for 1.2 mM uniformly ^13^C, ^15^N-labeled dimeric WT FlSp NT were recorded in 20 mM sodium acetate-d_3_, pH 5.5, 20 mM NaCl, 0.03% (w/v) NaN_3_, 10% D_2_O (v/v). For the assignment, 3D HNCA, CBCA(CO)NH, HNCACB, HN(CA)CO, HNHA, ^15^N-resolved NOESY-HSQC, ^13^C(aliphatic)-resolved NOESY-HSQC, and ^13^C(aromatic)-resolved NOESY-HSQC spectra were recorded. The spectra were processed using Topspin 4.0.7 (Bruker) and analyzed in CARA (freeware (40)). For comparison of structural fingerprints, 2D ^15^N-^1^H HSQC NMR spectra of WT FlSp NT, NT∗, NT_E79QE84QE119Q_, and NT_E79QE84QE119QE130Q_ were recorded. The ^15^N-labeled samples were prepared in either 20 mM sodium acetate-d_3_ and 20 mM NaCl, pH 5.5 or 20 mM sodium phosphate and 20 mM NaCl (FlSp NT∗) or 300 mM NaCl (FlSp NT_E79Q/E84Q/E119Q_ and NT_E79Q/E84Q/E119QE130Q_), pH 7.2 buffers (20, 21, 26).

For MW estimation 1D ^1^H-detected ^15^N relaxation experiments were performed. The ^15^N T_1_ and T_2_ relaxation data were recorded using the HSQC-based pulse sequences (41, 42) with delay times set at 20, 50, 100, 200, 300, 400, 600, 800, 1000, 1200, 1300, and 1500 ms for T_1_ and 16.96, 33.92, 50.88, 67.84, 84.80, 101.76, 135.68, 169.60, 203.52, 237.44, 271.36, and 339.2 ms for T_2_. T_1_ and T_2_ values were extracted by plotting the decay of integrated ^1^H^N^ intensities between δ ≈ 7.0 and 9.5 ppm using Topspin 4.0.7 (Bruker), and the curves were fitted with standard exponential equations using MestReNova 14.0.0 (Mestrelab). τ_c_ of the FlSp NT variants were calculated from the ^15^N T_1_/T_2_ ratio using following approximation:

$$\tau_{c}=\left(\sqrt{\frac{6T_{1}}{T_{2}}-7}\right)/4\pi \nu_{N}$$

where $\nu_{N}$ is the resonance frequency of ^15^N in Hz (43, 44). The MWs of proteins were estimated using the approximation that τ_c_ (in ns) is approximately 0.5 times the MW (in kDa).

For measuring diffusion coefficients, PFG-NMR experiments were run on the 600 MHz spectrometer at 298 K. The experiments were carried out using the Bruker pulse sequence stebpgp1s19 and were set up using the Bruker diffusion ordered spectroscopy macro employing a linear increase in gradient field strength from 2% to 98% over 16 1D experiments. The diffusion time, d20 (Δ), and the gradient pulse length, p30 (δ) were set to 100 ms and 2.2 ms respectively. Water suppression was achieved using Watergate 3-9-19. The self-diffusion coefficients (D_s_) were calculated by plotting the decay of integrated ^1^H signal intensity between 6.0 and 9.5 ppm and by fitting the curves with standard exponential equations using MestReNova 14 (Mestrelab).

### NMR structure calculation

Automated peak picking of the three NOESY spectra was performed using UNIO-ATNOS/CANDID 2.0.3 (45, 46). Torsion-angle restraints were obtained from the chemical shifts using TALOS (47). Structure calculation using the three peak lists, TALOS torsion angle restraints and chemical shifts as input was performed using CYANA 2.1 (48). The calculation involved seven iterations of automated NOE assignments with the routine CANDID (46) followed by simulated annealing procedure with 10,000 torsion-angle dynamics steps per conformer. Hundred conformers were calculated in each cycle. To increase the number of automatically assigned NOESY cross peaks and improve structural convergence, the structure calculation was started in the first cycle from a homology model built from *E. australis* MaSp NT monomer (PDB ID: 2LPJ) or dimer (PDB ID: 3LR2) as template instead of starting from random conformers. Unambiguous distance restraints were obtained in the last cycle of this automated procedure. The 20 conformers (Fig. S2) with the lowest final target function values were energy-minimized in a water shell using the program CNS 1.2 (49), and their coordinates were deposited in the PDB (monomer PDB ID: 7A0I and dimer PDB ID: 7A0O). Table S1 summarizes input for the structure calculations and structural statistics about the energy-minimized NMR structures of monomeric and dimeric FlSp NT.

### X-ray crystallography

The crystals were obtained by sitting drop vapor technique by mixing 1 μl of protein (10 mg/ml in 20 mM Na acetate, 10 mM NaCl, pH 5.5) with 1 μl of precipitant (0.2 M MgCl_2_, 0.1 M Bis-Tris pH 5.5, 25% PEG 3350). The crystals were flash-frozen in liquid nitrogen. Data were collected remotely at BioMAX beamline of MAXIV synchrotron. The crystals belonged to space group P2_1_ and diffracted to about 1.8 Å resolution. Data were processed by iMosflm (50) and scaled by Scala (51) of CCP4 suite (52). Initially, we tried to solve the structure by molecular replacement; however, we failed after many trials. Therefore, we produced Se-Met–labeled protein and collected anomalous data from obtained crystals. The structure was solved by single-wavelength anomalous diffraction, using program Phaser (53) SAD pipeline. The initial model was built automatically with Buccaneer (54). The model was corrected manually in Coot (55) and refined by Refmac (56). Water molecules were picked automatically in Coot, followed by several model improvement and refinement cycles. Data collection, refinement, and validation statistics are shown in Table S2.

### CD spectroscopy

Experiments were performed on a Chirascan V100 CD spectrometer (Applied Photophysics Limited) using 300 μl cuvettes with 1 mm path length. For all measurements, the proteins were diluted to 10 μM in 20 mM sodium phosphate buffer at pH 5.5 or pH 8.0. Spectra were recorded from 260 nm to 185 nm, first at 25 °C, then after heating to 95 °C and finally after cooling down to 25 °C. For each temperature, four scans were recorded to calculate an average spectrum. To evaluate the stability of the proteins against heat-induced denaturation, temperature scans from 25 °C to 105 °C with 1 °C steps were performed while the CD at 222 nm was monitored.

## Data availability

Requests for further data should be directed to Nina Kronqvist (nina.kronqvist@ki.se) or Kristaps Jaudzems (kristaps.jaudzems@osi.lv). The FlSp NT monomer solution structure at pH 7.2, dimer solution structure at pH 5.5, and dimer crystal structure have been deposited to the PDB with the accession codes 7A0I, 7A0O and 7OOM, respectively.

## Supporting information

This article contains supporting information.

## Conflict of interest

The authors declare that they have no conflicts of interest with the contents of this article.
