# Supplementary material for: The dimerization mechanism of the N-terminal domain of spider silk proteins is conserved despite extensive sequence divergence
Source: J Biol Chem. 2022 Apr 7;298(5):101913. doi: 10.1016/j.jbc.2022.101913 (PMC9097459; doi:10.1016/j.jbc.2022.101913)
Supplement: Supplemental Figures S1–S8 and Tables S1, S2 [file mmc1.pdf]

The dimerization mechanism of the N-terminal domain of spider silk proteins is conserved despite extensive sequence divergence

Médoune Sarr, Kristine Kitoka, Kellie-Ann Walsh-White, Margit Kaldmäe, Rimants Metlāns, Kaspar Tārs, Alessandro Mantese, Dipen Shah, Michael Landreh, Anna Rising, Jan Johansson, Kristaps Jaudzems, Nina Kronqvist

Supporting information

Figure S1 – S8

Table S1 – S2

Figure S1: Sequence alignment of NT domains of different spidroins. The characterized NTs are shown by a red arrow. The alignment was performed using Geneious Alignment and contains the following amino acid sequences of NT: AcSp from *Araneus diadematus* (Genbank AWK58688.1), AcSp from *Araneus ventricosus* (Genbank AUH99620), AcSp from *Argiope argentata* (Genbank AHK09813), AcSp from *Argiope aurantia* (Genbank AHK09770), AcSp from *Argiope trifasciata* (Genbank AHK09781), AcSp from *Latrodectus geometricus* (Genbank AFX83566), AcSp from *Latrodectus hesperus* (Genbank AFX83557), AcSp from *Nephila clavipes* (Genbank AWK58691), AcSp from *Parasteatoda tepidariorum* (Genbank AWK58692), AcSp from *Steatoda grossa* (Genbank AWK58693), FlSp from *Araneus diadematus* (GenBank AWK58735), FlSp from *Argiope argentata* (GenBank AWK58731), FlSp from *Latrodectus hesperus* (GenBank AWK58736), FlSp from *Nephila clavipes* (GenBank PRD27227), FlSp from *Parasteatoda tepidariorum* (GenBank AWK58739), FlSp from *Steatoda grossa* (GenBank AWK58740), MaSp from *Agelenopsis aperta* (GenBank ADM14324), MaSp from *Argyroneta aquatica* (GenBank AVH80563), MaSp from *Cybaeus angustiarum* (GenBank AVH80556), MaSp from *Diguetia canities* (GenBank ADM14315), MaSp 1 from *Araneus diadematus* (GenBank AWK58624), MaSp 1 from Argiope argentata

(GenBank AWK58623), MaSp 1 from Cyrtophora moluccensis (GenBank AGQ04592), MaSp 1 from *Euprosthenops australis* (GenBank CAJ90517), MaSp 1 from Kukulcania hibernalis (GenBank ADM14314), MaSp 1 from *Latrodectus geometricus* (GenBank ABY67428), MaSp 1 from *Latrodectus hesperus* (GenBank ABR68856), MaSp 1 from *Latrodectus mactans* (GenBank ADO78764), MaSp 1 from *Nephila clavipes* (GenBank ACF19411), MaSp 1 from *Parasteatoda tepidariorum* (GenBank AWK58631), MaSp 1 from *Steatoda grossa* (GenBank AWK58634), MaSp 2 from *Araneus diadematus* (GenBank AWK58651), MaSp 2 from *Argiope argentata* (GenBank AWK58646), MaSp 2 from *Argiope bruennichi* (GenBank AFN54363), MaSp 2 from *Argiope trifasciata* (GenBank AAZ15371), MaSp 2 from *Latrodectus geometricus* (GenBank ABY67417), MaSp 2 from *Latrodectus hesperus* (GenBank ABR68858), MaSp 2 from *Nephila clavipes* (GenBank AWK58654), MaSp 2 from *Nephila inaurata madagascariensis* (GenBank AAZ15322), MiSp from *Araneus diadematus* (GenBank AWK58672), MiSp from *Araneus ventricosus* (GenBank AFV31615), MiSp from *Argiope argentata* (GenBank AWK58671), MiSp from *Latrodectus geometricus* (GenBank ARA91186), MiSp from *Latrodectus hesperus* (GenBank ARA91182), MiSp from *Latrodectus tredecimguttatus* (GenBank ARA91189), MiSp from *Metepeira grandiosa* (GenBank ADM14328), MiSp from *Nephila clavipes* (GenBank AWK58680), MiSp from *Parasteatoda tepidariorum* (GenBank ARA91217), MiSp from *Steatoda grossa* (GenBank AWK58683), MiSp from *Uloborus diversus* (GenBank ADM14326), PiSp from *Araneus diadematus* (GenBank AWK58658), PiSp from *Argiope argentata* (GenBank AQR58363), PiSp from *Argyroneta aquatica* (GenBank AVH80558), PiSp from *Latrodectus hesperus* (GenBank AWK58659), PiSp from *Nephila clavipes* (GenBank PRD25616), PiSp from *Steatoda grossa* (GenBank AWK58661), TuSp from *Agelenopsis aperta* (GenBank ADM14330), TuSp from *Araneus ventricosus* (GenBank ASO67373), TuSp from *Argiope argentata* (GenBank ATW75951), TuSp from *Argiope bruennichi* (GenBank BAE86855), TuSp from *Latrodectus hesperus* (GenBank ABD24296), TuSp from *Nephila antipodiana* (GenBank ACI23395), TuSp from *Nephila clavata* (GenBank BAE54451), TuSp from *Nephila clavipes* (GenBank PRD35275), TuSp from *Steatoda grossa* (GenBank AWK58644).

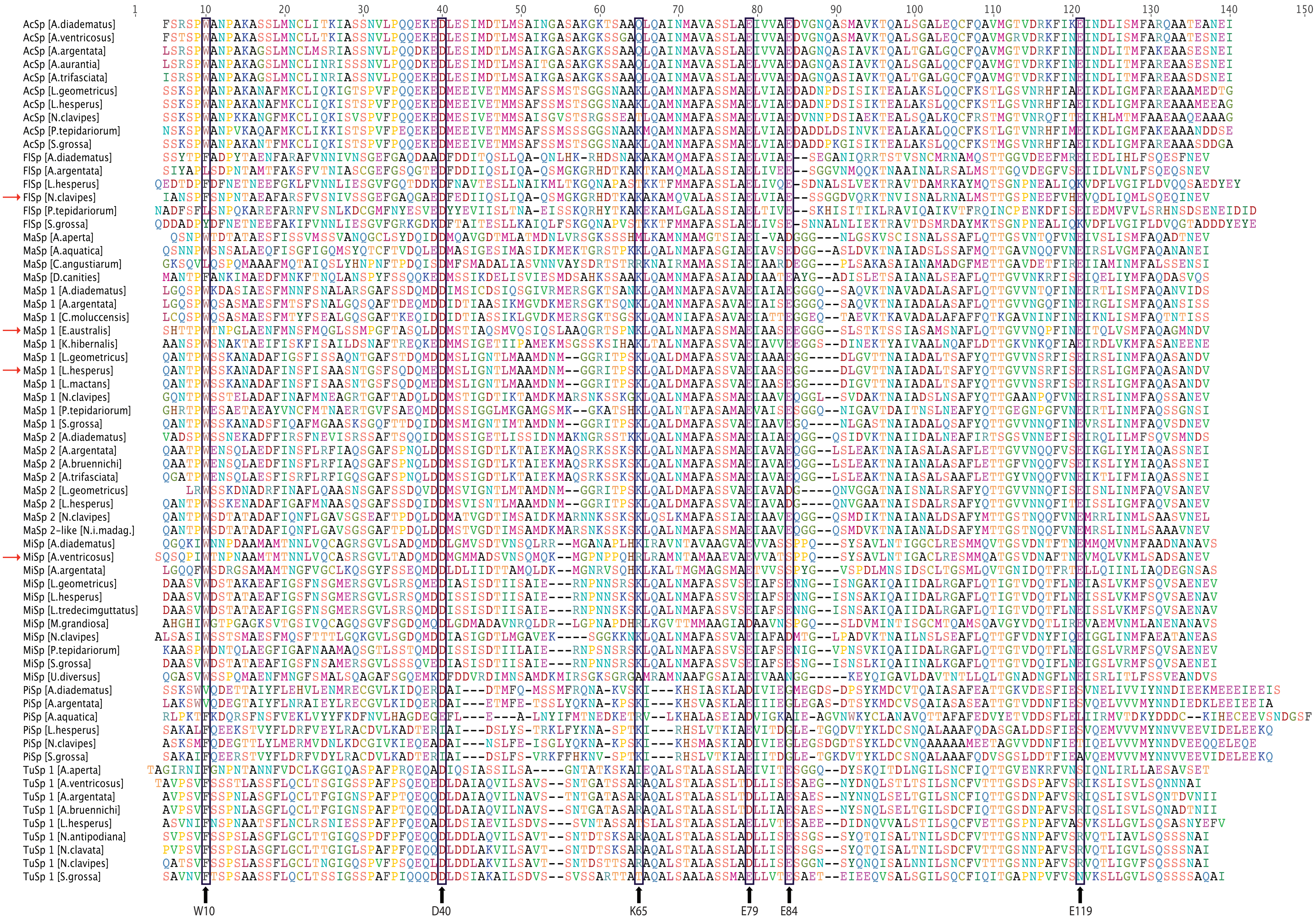
1
10
20
30
40
50
60
70
80
90
100
110
120
130
140
150
AcSp [A.diadematus]
AcSp [A.ventricosus]
AcSp [A.argentata]
AcSp [A.aurantia]
AcSp [A.trifasciata]
AcSp [L.geometricus]
AcSp [L.hesperus]
AcSp [N.clavipes]
AcSp [P.tepidariorum]
AcSp [S.grossa]
FlSp [A.diadematus]
FlSp [A.argentata]
FlSp [L.hesperus]
FlSp [N.clavipes]
FlSp [P.tepidariorum]
FlSp [S.grossa]
MaSp [A.aperta]
MaSp [A.aquatica]
MaSp [C.angustiarum]
MaSp [D.canities]
MaSp 1 [A.diadematus]
MaSp 1 [A.argentata]
MaSp 1 [C.moluccensis]
MaSp 1 [E.australis]
MaSp 1 [K.hibernalis]
MaSp 1 [L.geometricus]
MaSp 1 [L.hesperus]
MaSp 1 [L.mactans]
MaSp 1 [N.clavipes]
MaSp 1 [P.tepidariorum]
MaSp 1 [S.grossa]
MaSp 2 [A.diadematus]
MaSp 2 [A.argentata]
MaSp 2 [A.bruennichi]
MaSp 2 [A.trifasciata]
MaSp 2 [L.geometricus]
MaSp 2 [L.hesperus]
MaSp 2 [N.clavipes]
MaSp 2-like [N.i.madag.]
MiSp [A.diadematus]
MiSp [A.ventricosus]
MiSp [A.argentata]
MiSp [L.geometricus]
MiSp [L.hesperus]
MiSp [L.tredecimguttatus]
MiSp [M.grandiosa]
MiSp [N.clavipes]
MiSp [P.tepidariorum]
MiSp [S.grossa]
MiSp [U.diversus]
PiSp [A.diadematus]
PiSp [A.argentata]
PiSp [A.aquatica]
PiSp [L.hesperus]
PiSp [N.clavipes]
PiSp [S.grossa]
TuSp 1 [A.aperta]
TuSp 1 [A.ventricosus]
TuSp 1 [A.argentata]
TuSp 1 [A.bruennichi]
TuSp 1 [L.hesperus]
TuSp 1 [N.antipodiana]
TuSp 1 [N.clavata]
TuSp 1 [N.clavipes]
TuSp 1 [S.grossa]
W10
D40
K65
E79
E84
E119

Figure S2: Superposition of the 20 conformers with the lowest target functions. Superpositions of the polypeptide backbone heavy atoms of residues 11-55 and 61-130 for **(A)** the bundle of conformers representing the NMR structure of wt FlSp NT monomer, **(B)** the bundle of conformers representing the NMR structure of wt FlSp NT dimer and **(C)** the wt FlSp NT monomer (orange and dimer (green) structures showing the 20 conformers of Phe11 side chain.

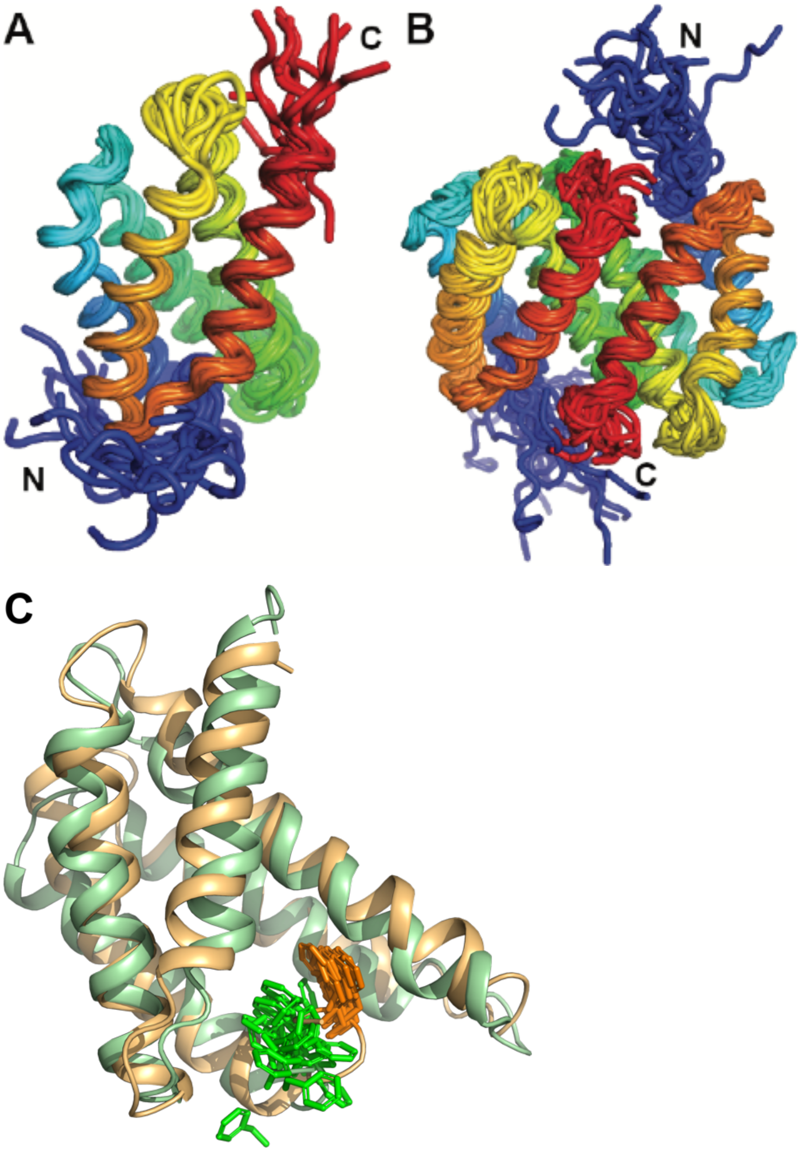

Figure S3: Size exclusion chromatography of wt FlSp NT. (A) Overlay of chromatograms at pH 5.5 (black) and at pH 8.0 (green). **(B)** Dimer to monomer ratio calculated from the apparent molecular weights of wt FlSp NT at pH 5.5 and pH 8.0, as determined from the elution volumes compared to a set of calibrants at each pH.

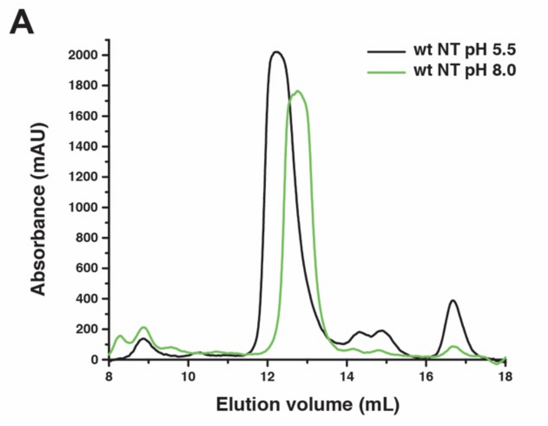

B

pH 8.0	pH 5.5	MW ratio
21.5 kDa	27.4 kDa	1.3

Figure S4: ESI-MS analysis of wt FlSp NT dimers at different concentrations. Spectra measured at pH 5.5 show predominantly dimers at concentrations ranging from 7 – 125 µM.

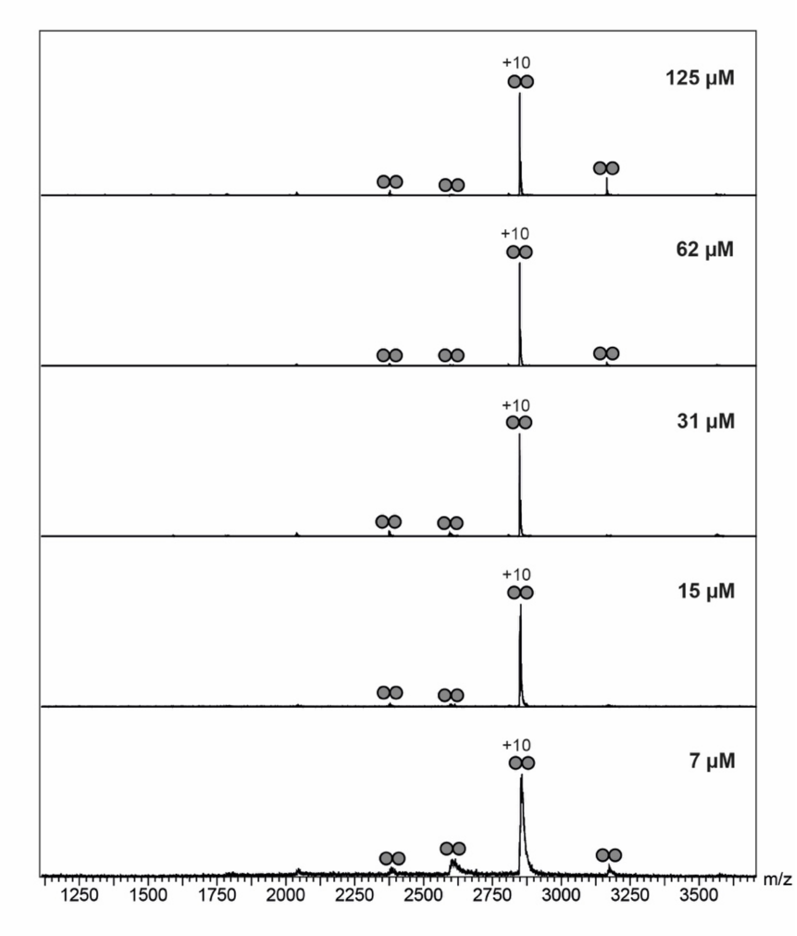

Figure S5: Dimerization studies of Phe to Trp mutants. Analysis was performed for NT_{Trp} (red) and NT^{*}_{Trp} (blue) by **(A)** size exclusion chromatography at pH 8.0 (solid lines) and at pH 5.5 (dashed lines) or by tryptophan fluorescence spectroscopy of **(B)** NT_{Trp} and **(C)** NT^{*}_{Trp}. The fluorescence was measured at pH 5.2 (brown), pH 5.6 (red), pH 6.0 (pink), pH 6.4 (orange), pH 6.8 (green), pH 7.2 (blue), pH 7.6 (gray) and pH 8.0 (black).

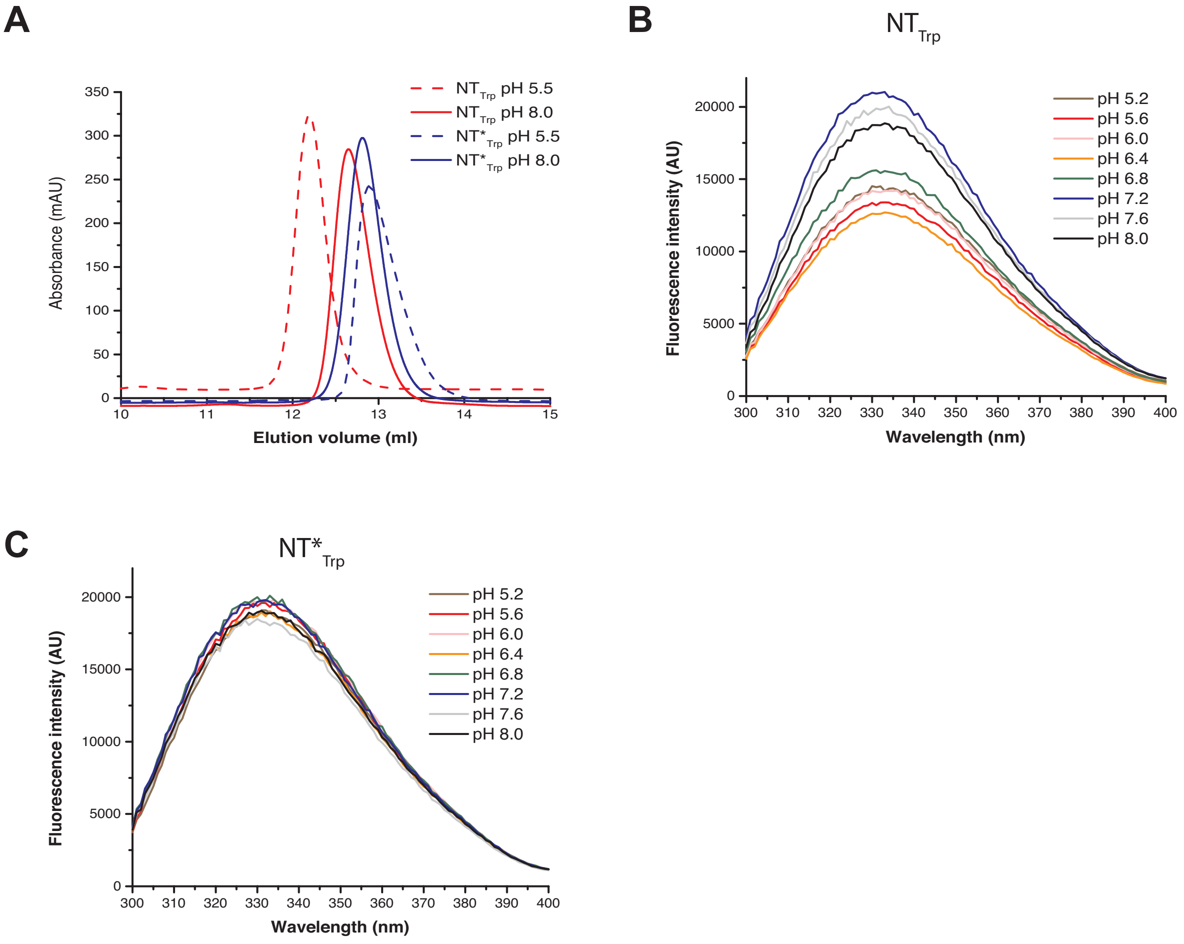

Figure S6: NMR analysis of wt FlSp NT and mutants. (A) Averaged backbone amide ^{1}H and ^{15}N chemical shift differences $\Delta\delta_{av} = \sqrt{(0.1 \Delta \delta_N)^2 + (\Delta \delta_H)^2}$ between wt FlSp NT at pH 7.2 and pH 5.5 (left panel). The chemical shift difference per residue (center panel) and the charge distribution of FlSp NT are displayed in surface representations. HSQC spectra of **(B)** FlSp NT* at pH 7.2 (green) and 5.5 (yellow), **(C)** FlSp NT$_{E79QE84QE119Q}$ at pH 7.2 (purple) and 5.5 (orange) and **(D)** FlSp NT$_{E79QE84QE119QE130Q}$ at pH 7.2 (black) and 5.5 (pink). **(E)** Estimation of the molecular weight of wt FlSp NT and FlSp NT mutants at pH 7.2 and pH 5.5 using ^{15}N NMR relaxation analysis. The table shows (from left to right) the ^{15}N NMR T_1 and T_2 relaxation times, the rotational correlation times (τ_c) and the estimated molecular weights (MW).

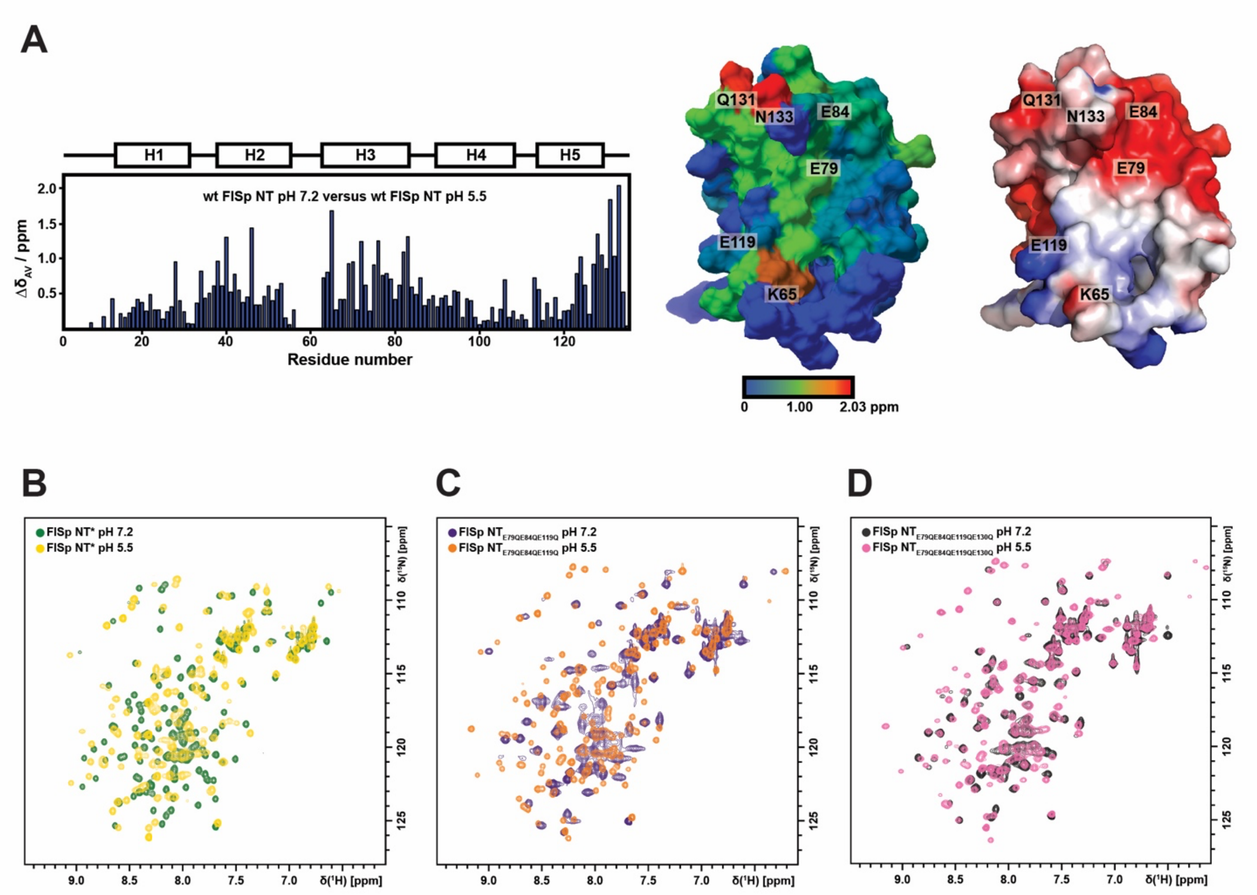

E

Protein	T_1 [ms]	T_2 [ms]	τ_c [ns]	MW [kDa]
wt FlSp NT pH 7.2	708	83.9	8.4	16.8
wt FlSp NT pH 5.5	848	64.2	13.2	26.4
FlSp NT* pH 7.2	693	96.1	7.2	14.4
FlSp NT* pH 5.5	784	78.8	9.9	19.8
FlSp NT$_{E79QE84QE119Q}$ pH 7.2	772	64.3	12.0	24.0
FlSp NT$_{E79QE84QE119Q}$ pH 5.5	955	70.0	13.6	27.3
FlSp NT$_{E79QE84QE119QE130Q}$ pH 7.2	1003	82.8	10.6	21.2
FlSp NT$_{E79QE84QE119QE130Q}$ pH 5.5	1001	67.4	11.9	23.7

Figure S7: Intensity decay profile for PFG-NMR self-diffusion measurements of wt NT at pH 7.2 and pH 5.5. The data have been fitted to an exponential equation using MestReNova 14.

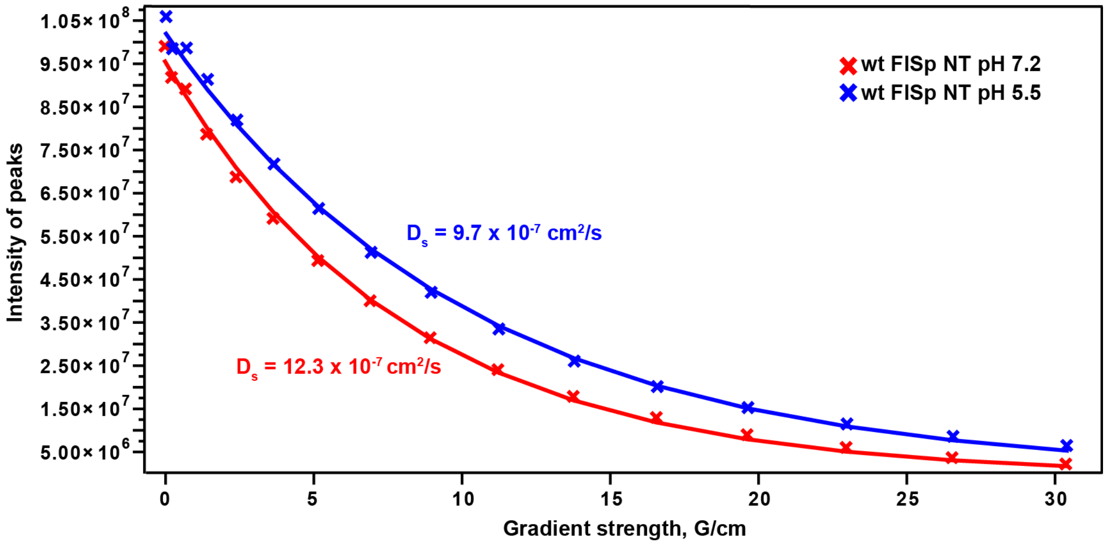

Figure S8: Refolding capacity and thermal stability measured with circular dichroism spectroscopy. **(A)** Spectra were measured in 20 mM sodium phosphate at 25°C (red), 95°C (black), and the ability to refold was evaluated from spectra after cooling the samples to 25°C (blue). The data are presented as a smoothed average of five measurements. **(B)** The thermal stability of wt NT (black), NT* (red) and $NT_{E79QE84QE119Q}$ (blue) was assessed by temperature-induced denaturation. Ellipticity at 222 nm was measured as a function of temperature. The respective T_m of the NT variants are indicated between brackets.

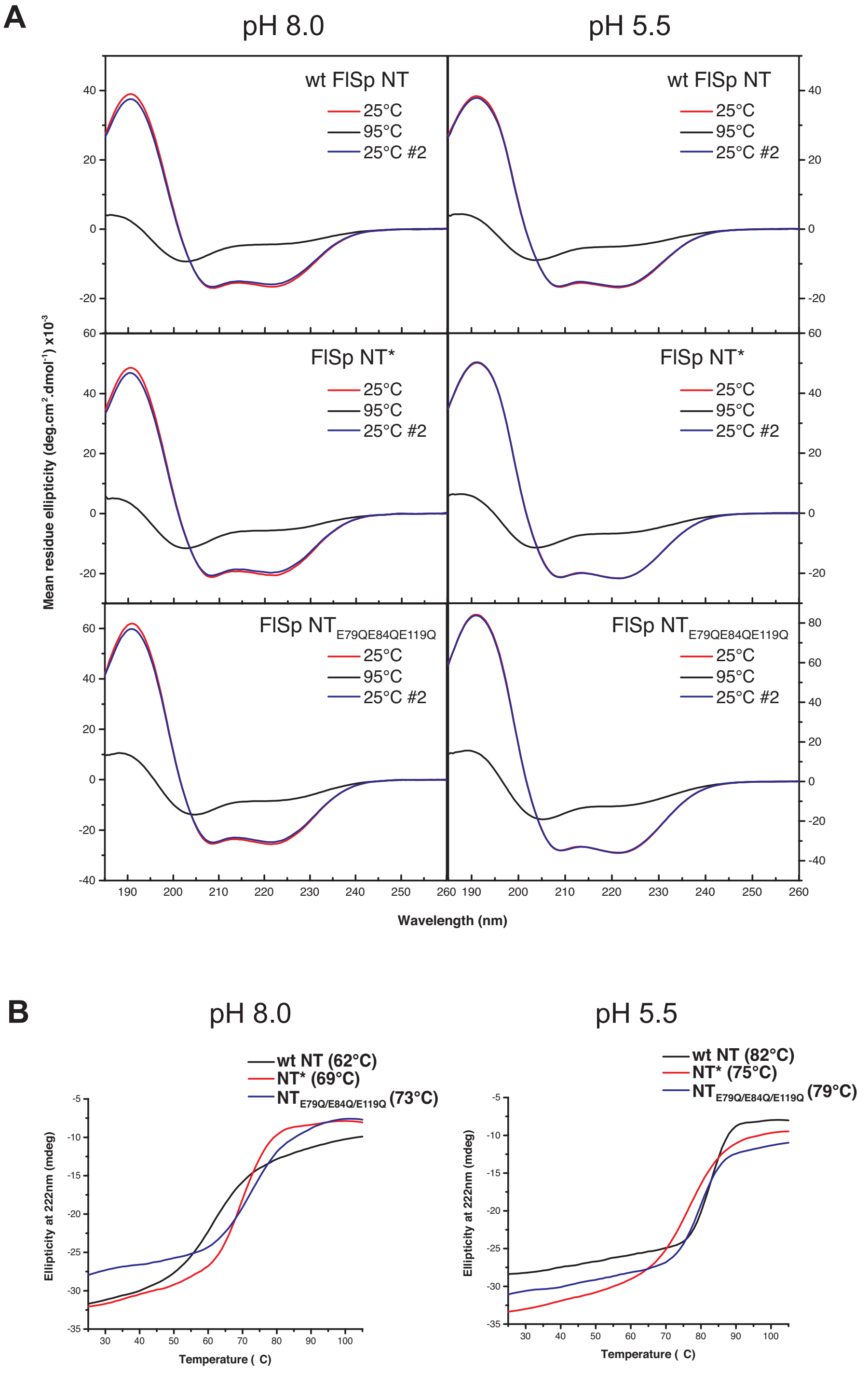

Table S1. Input for structure calculations and structural statistics of the NMR structures of FlSp NT monomer and dimer at pH 7.2 and pH 5.5, respectively.

**Quantity**	**Monomer at pH 7.2**[a]	**Dimer at pH 5.5**[a]
**Extent of chemical shift assignment**		
backbone H, ^{15}N, ^{13}Cα, Hα atoms	92.1%[b]	99.6%
non-labile side chain protons	97.2%	98.6%
**NOE upper distance limits**	1387	2300
intra-residual (|i-j|=0)	466	838
short-range	365	650
medium-range	291	368
long-range	265	408
intermolecular	-	36
**Residual NOE violations**		
number ≥ 0.1 Å	9 ± 3	20 ± 3
maximum [Å]	0.22 ± 0.05	0.27 ± 0.09
**PARALLHDG energies [kcal/mol]**		
total	-4951 ± 76	-10715 ± 174
van der Waals	-455 ± 25	-1027 ± 32
electrostatic	-5332 ± 79	-11352 ± 159
**R.m.s.d. from mean coordinates [Å]**		
backbone (residues 11–55,61–130)[c]	0.74 ± 0.12	1.00 ± 0.14
all heavy atoms (residues 11–55,61–130)[c]	1.13 ± 0.09	1.41 ± 0.16
**Ramachandran plot statistics**		
most favored regions [%]	94.3	88.9
additional allowed regions [%]	5.3	10.6
generously allowed regions [%]	0.3	0.4
disallowed regions [%]	0.1	0.1

[a]Except for the top eight entries, average values and standard deviations for the 20 energy-minimized conformers are given.

[b]Missing assignments for the first 9 residues (including five N-terminal residues GSGNS stemming from the expression and purification tag) and residues 57-62 located in a loop between helices 2 and 3.

[c]For the dimer, values given are for both chains.

Table S2. X-ray data collection, processing and refinement statistics.

Data collection and processing	
X-ray source	MAX IV BEAMLINE BioMAX
Wavelength (Å)	0.9788
Space group	$P2_1$
Cell dimensions *a, b, c* (Å), β (°)	41.88, 58.89, 46.33, 106.65
Resolution limits (Å)	44.39-1.80 (1.90-1.80)[a]
Observations	100490 (6887) [a]
Unique reflections	18569 (1906)[a]
Completeness (%)	92.6 (66.0)[a]
R_{merge}	0.094 (0.324)[a]
⟨I/σI⟩	10.7 (2.0)[a]
Multiplicity	5.4 (3.6)[a]
**Refinement**	
R_{work}	0.186 (0.259)[a]
R_{free}	0.2173 (0.3390)[a]
Average B factor (Å^2)	26.3590
⟨B⟩ from Wilson plot (Å^2)	23.9
No. atoms	
Protein	1932
Water	71
Ion	0
R.m.s. deviations from ideal values	
Bond lengths (Å)	0.016
Bond angles (°)	1.9
Outliers in Ramachandran plot (%)	0
PDB code	7OOM

[a] Values in brackets refer to the highest resolution shell.
